# Synthesis of Acridines through Alkyne Addition to Diarylamines

**DOI:** 10.3390/molecules23112867

**Published:** 2018-11-03

**Authors:** Kristen E. Berger, Grant M. McCormick, Joseph A. Jaye, Christina M. Rozeske, Eric H. Fort

**Affiliations:** Department of Chemistry, University of St. Thomas, 2115 Summit Avenue, St. Paul, MN 55115, USA; berger46@purdue.edu (K.E.B.); grant.mccormick@unmc.edu (G.M.M.); jaye4028@ucla.edu (J.A.J.); christina.rozeske@gmail.com (C.M.R.)

**Keywords:** cyclization, acridine, one-pot, terminal-alkyne, polycycles

## Abstract

A new synthesis of substituted acridines is achieved by palladium-catalyzed addition of terminal acetylenes between the aryl rings of bis(2-bromophenyl)amine. By including a diamine base and elevating the temperature, the reaction pathway favors the formation of acridine over a double Sonogashira reaction to form bis(tolan)amine. This method is demonstrated with several aryl-alkynes and alkyl-alkynes.

## 1. Introduction

New approaches to the synthesis of aromatic molecules have proven repeatedly to yield more than just new structures. Advances in the understanding of pyrolysis have opened the door to fullerenes and carbon nanotubes and their rational synthesis [1,2,3,4,5]. Study of ring closures and the packing of small molecules have bolstered the reputation and uses of grapheme [6,7,8]. Our group has focused on the ring-closure mechanisms of aromatic molecules, including the changes induced when boron and nitrogen are introduced into the carbon framework [9,10]. In synthesizing new precursors for study, well-known cross-coupling pathways were diverted to produce a variety of polycyclic aromatic molecules, particularly 9-substituted acridines. This approach to acridine synthesis has not been observed previously, and it holds a great deal of potential for future synthetic and mechanistic insight.

Acridines are characterized by the linear fusing of three aromatic 6-membered rings with a nitrogen atom included in the center ring [11]. Acridines were first isolated from coal tar by Graebe in 1870 [12]. The ability of acridine to bind DNA has made it a target for anticancer and antimicrobial uses, but it is most often found in organic dyes and staining agents for microscopy [13,14,15]. More recently, acridines have been investigated for their electronic properties and as photo-redox catalysts [16]. 

This work began upon the observation that the microwave reaction of phenylacetylene with bis(2-bromophenyl)amine (**3**) in the presence of a diamine solvent resulted in a significant yield of an unknown molecule. Isolation and characterization of this structure revealed that the reaction was producing 9-benzylacridine as the major product, as well as other aromatic products such as carbazole. Acridines are typically synthesized by electrophilic aromatic substitution of pendant carboxylic acid derivatives or by adding groups to the edges of smaller rings [17,18,19,20,21,22,23]. Both of these methods typically require further processing to aromatize the acridine ring following closure. Though some tandem processes have been shown previously, none incorporates alkynes in a manner similar to this work [24,25,26,27]. Two elegant C-H activation papers have been produced, both beginning with already-formed acridine [28,29]. Since alkynes are carbon-rich, they provide an especially attractive departure point into the aromatic chemistry [30]. The aryl-bromides in our system appeared to be providing an avenue for coupling and cyclization at the terminal carbon of the phenylacetylene, rather than proceeding to a second cross-coupling reaction with an additional alkyne. The diaryl amine route is a fast and versatile route for adding complexity. Though the reaction described below was first observed in a microwave, it also proceeds with traditional heating. Several methods have previously used microwave chemistry to produce acridines, but no similar pathway to benzylacridines can be found in the literature. The work described below explores the reaction in detail in order to elucidate its versatility [31,32].

## 2. Results

**Figure 1:**
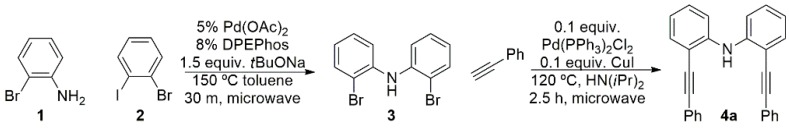
Cross-coupling reactions to produce precursor **3** and bis(tolan)amine (**4a**) [34,35].

The precursor bis(2-bromophenyl)amine (**3**) was synthesized in 68% yield from 2-bromoaniline (**1**) and bromoiodobenze (**2**) by a palladium-catalyzed cross coupling reaction in the presence of bis(2-diphenylphosphino)phenyl)ether (DPEPhos) (Figure 1) [33]. This system allows for a nearly complete reaction between the iodine and the aniline groups, with little evidence for homocoupling between halides or bromo-iodo mixtures in the products. The dibromo product **3** is converted to bis(tolan)amine (**4a**) in 63% yield by a double Sonogashira cross-coupling reaction with phenyl-acetylene and using diisopropylamine as a solvent. 

In an effort to increase yields, diisopropylamine was replaced with ethylene diamine and the temperature was raised. The results showed that the presence of the di-aryl amine and two bromine atoms makes **3** a multi-faceted molecule for synthesis. The amine group can be further functionalized by Buchwald-Hartwig-type reactions, the halides are excellent handles for cross couplings, and inter- and intra-molecular homocoupling reactions are possible as well [34]. With the diamine solvent, bis(tolan)amine was no longer the major product of the reaction. Instead, 9-benzylacridine (**5a**) was present with small amounts of **4a** and carbazole (**6**) from homocoupling of the ortho-bromides (Table 1).

The reaction was screened at varying temperatures with one equivalent of phenyl acetylene to optimize for acridine. Though 180 °C did not have the highest yield of acridine, it appeared that bis(tolan)amine **4a** and carbazole **6** products were minimized. This vastly simplified purification by shutting down other mechanistic pathways. The equivalents of alkyne, solvents, and substituents were varied. Yields of the acridine increased with more alkyne, while the byproducts did not seem to become more abundant. Ethylene diamine was replaced with tetramethylethylene diamine (TMEDA) to determine if the hydrogen atoms of the amine solvent were required, and the acridine yield remained comparable to the ethylene diamine. Electron-rich and -poor terminal alkynes, including alkyl substituted alkynes, all produced acridine in varying amounts. A range of 9-substituted products were produced, including benzyl (**5a**), 4-methylbenzyl (**5b**), 4-methoxybenzyl (**5c**), 4-fluorobenzyl (**5d**), heptyl (**5e**), and 4-phenylbutyl (**5f**). Though electron-rich substituents produced higher yields, it was possible to obtain acridines with 4-fluorophenylacetylene, and even alkynes with no conjugated aromatic groups, such as octyne and 5-phenyl-1-pentyne. The major side-products remained carbazoles from intramolecular homocoupling of the halides, as well as a variety of products with the terminal alkyne adding to the amine or alkyne degradation products, though all of these to a lesser extent than the formation of the acridine. 

The examples above all used microwave heating for the synthesis. Few examples of acridine synthesis in the literature use microwave reactors. [22,31,32] To ensure the reaction results were not related to the heat source, the synthesis of molecules **5a**–**f** were conducted under the same reaction conditions with conventional heating in round bottomed flasks at 180 °C in an oil bath. The results were consistent with the microwave reactions and had similar yields.

## 3. Discussion

**Scheme 1:**
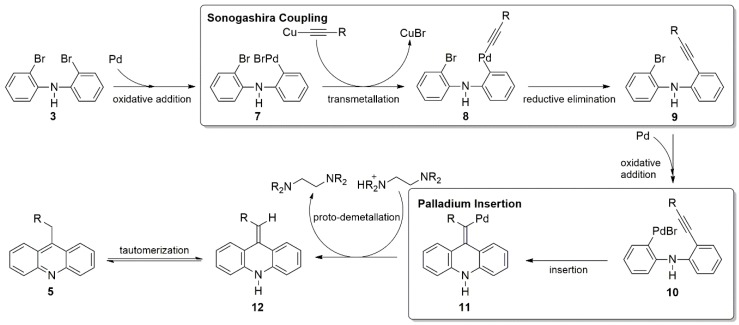
Proposed pathway for acridine formation. Catalytic cycles abbreviated for clarity.

Though mechanistic studies are ongoing, it is predicted that the terminal alkyne initially reacts with bis(2-bromophenyl)amine through a Sonogashira coupling to produce **9**. This would place the alkyne in proximity to the second bromide, where it can undergo an intramolecular cyclization at the terminal carbon via palladium insertion to give structure **11**. The conversion from **10** to **11** in the mechanism would be the critical step favoring acridine. The palladium must undergo an intramolecular reaction rather than transmetallation to facilitate another Sonogashira reaction. It is hypothesized that the presence of the diamine favors the intramolecular reaction, though further work is required to understand the nature of the preference. This process closes the diarylamine into a three-ring system. Proto-demetallation of the palladium produces **12** with a vinylic hydrogen between the two ring systems. This loss of palladium has been observed previously in the synthesis of indoles [35]. Tautomerization, driven by aromatization, would produce the acridine products (**5**) by installing a proton in the benzylic position while deprotonating the aryl amine. (Scheme 1). It should be noted that **9** has not been isolated, and an alternate pathway may exist where **8** is converted directly to **11**. This too would suppress the formation of **4**. Study of these potential pathways is ongoing.

The presence of the diamine ligand, HBr, produced in the reaction, and the driving force toward aromaticity likely favor the acridine formation. All reactions are degassed and run in the microwave, away from ambient light. These conditions prevent air oxidation, as well as any opportunity for the diarylamine or acridine to perform photoredox reactions.

## 4. Materials and Methods 

Reagents and anhydrous solvents were purchased from Sigma-Aldrich (St. Louis, MO, USA) or Alfa Aesar (Tewksbury, MA, USA). All other chemicals were used without purification unless otherwise noted. NMR spectra were acquired on a JEOL 400 MHz NMR spectrometer (JEOL, Peabody, MA, USA) with chloroform-*d*_1_ (δ_H_ = 7.26 ppm δ_C_ = 77.23 ppm) or acetone-*d*_6_ (δ_H_ = 2.05 ppm δ_C_ = 206.26 ppm). All coupling constants were generated by the MestReNova (Version 11.0, Mestrelab, Santiago de Compostela, Spain) program and were uncorrected. Chromatography was performed with Biotage KP-SIL™ (Biotage USA, Charlotte, NC, USA) or KP-NH™ (Biotage USA, Charlotte, NC, USA) cartridges or Silicycle silica gel (porosity = 60 Å, particle size 40–63 μm). Microwave reactions were run on the Discover Microwave System by CEM Corporation (Matthews, NC, USA). HRMS was acquired with an Agilent 7200 GC/QTOF (Agilent Technologies, Santa Clara, CA, USA) or Bruker Bio TOF II (Bruker, Billerica, MA, USA) with Electrospray Ionization.

### 4.1. General Procedure for the Synthesis of 9-Substituted Acridine (***5a**–**f***) Derivatives

#### 4.1.1. Microwave Heating

To a flame-dried 35 mL microwave vial with a teflon coated cap, 25 mL of ethylene diamine was added via syringe and degassed with nitrogen for 20 min. To this vessel was added 504 mg (1.5 mmol) of bis(2-bromophenyl)amine (**3**), 66 mg (0.30 mmol) of copper iodide, and 219 mg (0.30 mmol) of bis(triphenylphosphine)palladium dichloride sequentially while stirring under nitrogen. Next, 3.0 mmol of aryl acetylene was added dropwise via syringe.

The reaction flask was heated for 1.5 h at 180 °C in a microwave reactor. Upon completion, the reaction was then poured over ice, extracted into dichloromethane (3 × 35 mL), and the combined organic layers were washed with water three times before purifying on an amine-treated KP-NH™ silica flash column using hexanes and ethyl acetate as eluent. Fractions primarily include bis(tolan)amines (**4a**–**f**), 9-substituted acridines (**5a**–**f**), and carbazole (**6**), depending on conditions.

#### 4.1.2. Conventional Heating

To a flame-dried 150 mL pressure vessel, 25 mL of ethylene diamine was added via syringe and degassed with nitrogen for 20 min. To this vessel was added 504 mg (1.5 mmol) of bis(2-bromophenyl)amine (3), 66 mg (0.30 mmol) of copper iodide, and 219 mg (0.30 mmol) of bis(triphenylphosphine)palladium dichloride sequentially while stirring under nitrogen.

Next, 3.0 mmol of aryl acetylene was added dropwise via syringe. The reaction flask was heated for 1.5 h in an oil bath set to 180 °C. Upon completion, the reaction was then poured over ice, extracted into dichloromethane (3 × 35 mL), and the combined organic layers were washed with water three times before purifying on an amine treated KP-NH™ silica flash column using hexanes and ethyl acetate as eluent. Fractions include primarily bis(tolan)amines (**4a**–**f**), 9-substituted acridines (**5a**–**d**), and carbazole (**6**), depending on conditions. See supporting information for further details.

## 5. Conclusions

The one-pot synthesis of 9-substituted acridines from terminal alkynes and bis(2-bromophenyl)amine occurs in the presence of a diamine solvent. In the absence of the diamine solvent, bis(tolan)amine can also be synthesized. This methodology provides rapid access to a variety of structures bearing both donating and withdrawing groups, and is possible with microwave or traditional heating. The reaction also produces carbazole and cross-coupling products in small amounts. Mechanistic work is ongoing.

## 6. Patents

A patent regarding this material has been filed. Fort, E.H.; Jaye, J.A.; McCormick, G.M.; Berger, K.E. Synthesis of Substituted Acridines US Patent US18/33,224, filed 17 May 2018.

## Figures and Tables

**Table 1:**
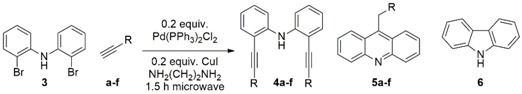
Production of 9-substituted acridines **5a**–**f**.

Entry	Alkyne (R)	Temp.	4/5/6 (%) ^4^
1 ^1^	**a** (Ph)	120 °C	7/14/3
2 ^1^	**a** (Ph)	140 °C	trace/9/7
3 ^1^	**a** (Ph)	160 °C	3/29/13
4 ^1^	**a** (Ph)	180 °C	trace/24/trace
5 ^1^	**a** (Ph)	200 °C	trace/30/7
6 ^2^	**a** (Ph)	180 °C	trace/53/6
7 ^2,3^	**a** (Ph)	180 °C	0/51/9
8 ^2^	**b** (p-MeC_6_H_4_)	180 °C	0/55/3
9 ^2^	**c** (p-MeOC_6_H_4_)	180 °C	trace/35/trace
10 ^2^	**d** (p-FC_6_H_4_)	180 °C	0/27/6
11 ^2^	**e** (CH_2_)_5_CH_3_	180 °C	0/19/3
12 ^2^	**f** (C_3_H_6_Ph)	180 °C	0/10/3

^1^ 1 eq. of alkyne; ^2^ 2 eq. of alkyne; ^3^ tetramethylethylenediamine; ^4^ All reported values are isolated yields; trace indicates observed in ^1^H-NMR and not isolated.

## Supplementary Materials

The following are available online, detailed synthetic procedures and characterization for molecules **4a** and **5a**–**f**.
