# Supplementary material for: Synthesis of Acridines through Alkyne Addition to Diarylamines
Source: Molecules. 2018 Nov 3;23(11):2867. doi: 10.3390/molecules23112867 (PMC6278640; doi:10.3390/molecules23112867)
Supplement: Supplementary file 1 [file molecules-23-02867-s001.pdf]

Supporting Information for

Synthesis of 9-substituted acridines from bis(bromophenyl)amine

Kristen E. Berger, Grant M. McCormick, Joseph A. Jaye, Christina M. Rozeske, and

Eric H. Fort*

University of St. Thomas, Department of Chemistry, 2115 Summit Avenue, St. Paul, MN 55115, United States of America

E-mail: ehfort@stthomas.edu

Telephone 1 651 962 5588

Fax 1 651 962 5201

Table of contents:

General Experimental……………………………………………………………..……S1
Synthesis of bis(bromophenyl)amine (**3**)……………………………………………..…...S2
Synthesis of bis(tolan)amine (**4a**)…………………………………………………...…..S2-3
General procedure for the synthesis of 9-substituted acridine (**5a-f**) derivatives………...….S3
Preparation of 9-(benzyl)acridine (**5a**)…...........……………………………………..S3-4
Preparation of 9-(4-methylbenzyl)acridine (**5b**)……………………….......…..............S3
Preparation of 9-(4-methoxybenzyl)acridine (**5c**)……………………………...…............S4
Preparation of 9-(4-fluorobenzyl)acridine (**5d**)…………………………....….............S4-5
Preparation of 9-heptylacridine (**5e**)……………………………………………………...…S5
Preparation of 9-(4-phenylbutyl)acridine (**5f**)………………………..……….................S5
Spectra for all molecules……………………………………………………….………...S6-S21

General Experimental:

Reagents and anhydrous solvents were purchased from Sigma-Aldrich or Alfa Aesar. All other chemicals were used without purification unless otherwise noted. NMR spectra were acquired on a JEOL 400 MHz NMR spectrometer with chloroform-d_1 (δ_H = 7.26 ppm δ_C = 77.23 ppm) or acetone-d_6 (δ_H = 2.05 ppm δ_C = 206.26 ppm). All coupling constants were generated by the MestReNova program and were uncorrected. Chromatography was performed with Biotage KP-SIL™ or KP-NH™ cartridges or Silicycle silica gel (porosity = 60 Å, particle size 40-63 µm). Microwave reactions were run on the Discover Microwave System by CEM Corporation. HRMS was acquired with an Agilent 7200 GC/QTOF or Bruker Bio TOF II with Electrospray Ionization.

Synthesis of bis(bromophenyl)amine (3):

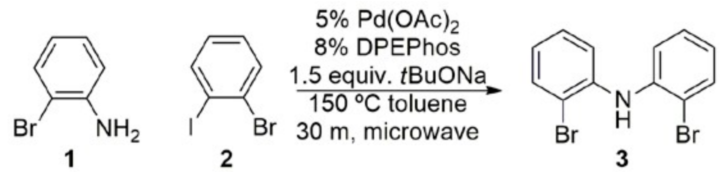

To a flame dried 35 mL microwave vial with a teflon cap, 16 mL of anhydrous toluene was added followed by 3.18 g (33.1 mmol) of sodium *tert*-butoxide then 2.6 mL (23 mmol) of 2-bromoaniline (**1**) and 3.0 mL (23 mmol) of 2-bromoiodobenzene (**2**) dropwise and stirred. The ligand DPEPhos, 103 mg (0.190 mmol), and 29.8 mg (0.133 mmol) $Pd(OAc)_2$ were added then heated 30 minutes at 150 °C in a microwave reactor. The product was extracted with 3 x 35 mL of dichloromethane and the combined organic layer was washed with water (3 x 150mL). The organic layer was dried over magnesium sulfate and concentrated *in vacuuo*. The resulting product was recrystallized from ethanol to yield white or gray needle-like crystals in 68 % yield.

MP 59.8-60.7 °C **^{1}H-NMR** (400 MHz, $CDCl_3$) δ: 7.58 (dd, J = 8.0 Hz, 1.6 Hz, 2H), 7.30 (dd, J = 8.0 Hz, 1.6 Hz, 2H), 7.22 (td, J = 8.4 Hz, 1.2 Hz, 2H), 6.84 (td, J = 7.6 Hz, 2.0 Hz, 2H), 6.44 (s, 1H) **^{13}C-NMR** (100 MHz, $CDCl_3$) δ: 140.13, 133.39, 128.23, 122.67, 118.08, 114.38 **HRMS** (EI) calc. for $C_{12}H_9Br_2N$ 324.9102, found 324.9094 **UV-Vis** (CH_2Cl_2) λ_{max} (ε, $cm^{-1}M^{-1}$) 287 (39000)

Synthesis of bis(tolan)amine (4a):

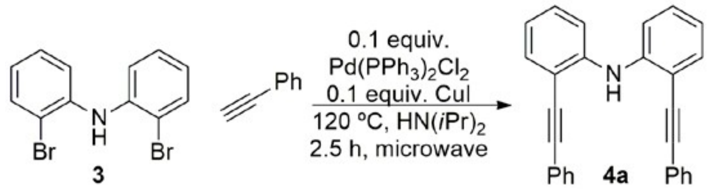

To a flame dried 35mL microwave vial with a teflon cap, 25 mL of diisopropylamine was added via syringe and degassed with nitrogen for ten minutes. To this vessel 510 mg (1.6 mmol) of bis(2-bromophenyl)amine (**3**), 140 mg (0.74 mmol) CuI, and 111 mg $Pd(PPh_3)_2Cl_2$ (0.16 mmol) were added sequentially while stirring under nitrogen. Subsequently 0.84 mL of phenyl acetylene was added dropwise via syringe over 30 min followed by heating for 2.5 hours at 120 °C in a microwave reactor. Upon completion, the reaction was poured over ice and extracted into dichloromethane (3 × 50mL). The combined organic layer was washed with water (3 × 100mL) then concentrated *in vacuuo*. The reaction was then purified using a KP-NH™ flash column using hexanes and ethyl acetate to produce a yellow solid in 63% yield.

MP 136.6-137.1 °C **^{1}H-NMR** (400 MHz, $CDCl_3$) δ: 7.53 (dd, J = 1.4 Hz, 7.7 Hz, 2H), 7.49 (d, J = 8.4 Hz, 2H), 7.38 (m, 5H), 7.28 (t, J = 7.3 Hz, 2H), 7.22-7.17 (m, 2H), 7.13 (t, J = 7.2 Hz, 4H), 6.90 (t, J = 7.6 Hz, 2H) **^{13}C-NMR** (100 MHz, $CDCl_3$) δ: 143.2, 132.9, 131.7, 129.4, 128.4, 128.3, 122.9, 120.1, 115.3, 112.2, 96.2, 85.6 **HRMS** (ESI) calc. for $C_{28}H_{19}NNa$ $[M + Na]^+$ 392.141519, found 392.1433 **UV-Vis** (CH_2Cl_2) λ_{max} (ε, $cm^{-1}M^{-1}$) 279 (160000), 312 (37000), 340 (44000)

General Procedure for the synthesis of 9-substituted acridine (5a-f) derivatives:

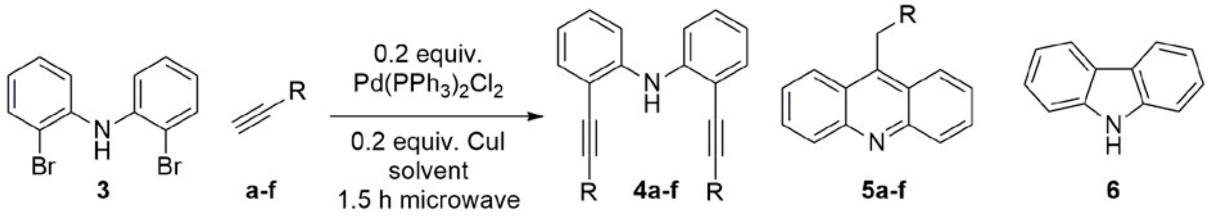

Microwave Heating:

To a flame dried 35 mL microwave vial with a teflon coated cap, 25 mL of ethylene diamine was added via syringe and degassed with nitrogen for 20 minutes. To this vessel was added 504 mg (1.5 mmol) of bis(2-bromophenyl)amine (**3**), 66 mg (0.30 mmol) of copper iodide, and 219 mg (0.30 mmol) of bis(triphenylphosphine)palladium dichloride sequentially while stirring under nitrogen. Next 3.0 mmol of aryl acetylene was added dropwise via syringe. The reaction flask was heated for 1.5 hours at 180 ºC in a microwave reactor. Upon completion, the reaction was then poured over ice, extracted into dichloromethane (3 x 35 mL) and the combined organic layers were washed with water three times before purifying on an amine treated KP-NH™ silica flash column using hexanes and ethyl acetate as eluent. Fractions include primarily bis(tolan)amines (**4a-f**), 9-substituted acridines (**5a-f**), and carbazole (**6**) depending on conditions.

Conventional Heating:

To a flame dried 150 mL pressure vessel, 25 mL of ethylene diamine was added via syringe and degassed with nitrogen for 20 minutes. To this vessel was added 504 mg (1.5 mmol) of bis(2-bromophenyl)amine (**3**), 66 mg (0.30 mmol) of copper iodide, and 219 mg (0.30 mmol) of bis(triphenylphosphine)palladium dichloride sequentially while stirring under nitrogen. Next 3.0 mmol of aryl acetylene was added dropwise via syringe. The reaction flask was heated for 1.5 hours in an oil bath set to 180 ºC. Upon completion, the reaction was then poured over ice, extracted into dichloromethane (3 × 35 mL) and the combined organic layers were washed with water three times before purifying on an amine treated KP-NH™ silica flash column using hexanes and ethyl acetate as eluent. Fractions include primarily bis(tolan)amines (**4a-f**), 9-substituted acridines (**5a-d**), and carbazole (**6**) depending on conditions.

Preparation of 9-benzylacridine (5a):

The general procedure for the synthesis of 9-substituted acridine derivatives above was followed with 0.33 mL (3.0 mmol) phenyl acetylene. The reaction produced a mustard yellow solid in 203 mg (53%) yield by microwave heating. Conventional heating produces 217 mg (55%) yield.

MP 160.6-163.6 °C **^{1}H-NMR** (400 MHz, $CDCl_3$) δ: 8.25 (t, J = 8.0 Hz, 4H), 7.77 (t, J = 4.0 Hz, 2H), 7.53 (td, J = 8.0 Hz,1.2 Hz 2H), 7.24-7.16 (m, 3H), 7.11 (d, J = 6.8 Hz, 2H), 5.03 (s, 2H) **^{13}C NMR** (100 MHz, $CDCl_3$) δ: 149.0, 143.6, 139.5, 130.5, 130.0, 128.9, 128.3, 126.2, 126.2 125.8, 124.9, 33.3 **HRMS (**ESI) calc. for $C_{20}H_{16}N$ 270.1283, found 270.1204 **UV-Vis** (CH_2Cl_2) λ_{max} (ε, $cm^{-1}M^{-1}$) 255 (120000), 280 (sh,5100), 343 (5700), 361 (8600), 387 (38000)

Preparation of 9-(4-methylbenzyl)acridine (5b):

The general procedure for the synthesis of 9-substituted acridine derivatives above was followed with 0.39 mL (3.0 mmol) 4-ethynyltoluene. The reaction produced a pale white solid in 236 mg (55%) yield. Conventional heating produces 184 mg (40%) yield.

MP 160.6-163.6 °C **^{1}H-NMR** (400 MHz, $CDCl_3$) δ: 8.25 (dd J = 8.9 Hz, 9.1 Hz, 4H) 7.77 (ddd, J = 1.2 Hz, 8.8 Hz, 8.8 Hz, 2H), 7.52 (ddd, J = 1.2 Hz, 8.8 Hz, 8.9 Hz, 2H), 7.01 (aa′bb′ J = 8.4 Hz, 8.4 Hz, 4.8 Hz, 4H), 4.99 (s, 2H), 2.26 (s, 3H) **^{13}C-NMR** (100 MHz, $CDCl_3$) δ: 149.04, 143.90, 136.42, 136.16, 130.49, 129.95, 129.52, 128.12, 126.16, 125.80, 124.94, 32.89, 21.11 **HRMS** (ESI) calc. For $C_{21}H_{18}N$ $[M+1]^+$ 284.1439, found 284.1431 **UV-Vis** (CH_2Cl_2) λ_{max} (ε, $cm^{-1}M^{-1}$) 255 (130000), 247 (sh,55000), 342 (6100), 362 (9100), 387 (3000)

Preparation of 9-(4-methoxybenzyl)acridine (5c):

The general procedure for the synthesis of 9-substituted acridine derivatives above was followed with 0.39 mL (3.0 mmol) 4-ethynylanisole. The reaction produced a light cream colored solid in 155 mg (35%) yield. Conventional heating produces 162 mg (36%) yield.

MP 135.9-138.7 °C **^{1}H-NMR** (400 MHz, $CDCl_3$) δ: 8.25 (dd, J = 7.8 Hz, 8.0 Hz, 4H), 7.77 (ddd, J = .8 Hz, 8.5 Hz, 8.6 Hz, 2H), 7.53 (ddd, J = 1.1 Hz, 8.6 Hz, 8.5 Hz, 2H), 7.02 (d, J = 8.7 Hz, 2H), 6.75 (d, J = 8.8 Hz, 2H), 4.96 (s, 2H), 3.72 (s, 3H) **^{13}C-NMR** (100 MHz, $CDCl_3$) δ: 158.2, 149.0, 144.0, 131.5, 130.5, 130.0, 129.2, 126.1, 125.7, 124.9, 114.2, 55.4, 32.4 **HRMS (**ESI) calc. for $C_{21}H_{18}NO$ 300.1388, found 300.1381 **UV-Vis** (CH_2Cl_2) λ_{max} (ε, $cm^{-1}M^{-1}$) 255 (430000), 247 (sh,180000), 344 (19000), 361 (28000), 387 (14000)

Preparation of 9-(4-fluorobenzyl)acridine (5d):

The general procedure for the synthesis of 9-substituted acridine derivatives above was followed with 0.35 mL (3.0 mmol) 1-ethynyl-4-fluorobenzene. The reaction produced a toffee-colored solid in 119 mg (27%) yield. Conventional heating produces 78 mg (18%) yield.

MP 163.6-165.9 °C **^{1}H-NMR** (400 MHz, DMSO) δ: 8.45 (d, J = 9.0 Hz, 2H), 8.18 (d, J = 8.5 Hz, 2H), 7.85 (dd, J = 7.7 Hz, 8.8 Hz, 2H), 7.64 (dd, J = 7.5 Hz, 8.8 Hz, 2H), 7.17 (dd, J = 8.8 Hz, 8.5 Hz, 2H), 7.04 (t, J = 8.8 Hz, 2H), 5.10 (s, 2H) **^{13}C-NMR** (100 MHz, $CDCl_3$) δ: 149.1, 143.2, 130.7, 130.0, 129.7, 129.6, 126.3, 125.7, 126.4, 125.7, 124.7, 115.8, 115.6, 32.5 **HRMS (**EI) calc. for $C_{20}H_{14}FN$ 287.1111, found 287.1108 **UV-Vis** (CH_2Cl_2) λ_{max} (ε, $cm^{-1}M^{-1}$) 255 (470000), 343 (23000), 360 (35000), 385 (16000)

Preparation of 9-heptylacridine (5e):

The general procedure for the synthesis of 9-substituted acridine derivatives above was followed with 0.45 mL (3.0 mmol) 1-octyne. The reaction produced a light red oil in 78 mg (19%) yield.

^{1}H-NMR (400 MHz, $CDCl_3$) δ: 8.23 (dd, J = 8.8 Hz, 8.8 Hz, 2H), 7.76 (dd, J = 7.7 Hz, 8.6 Hz, 2H), 7.56 (dd, J = 8.4 Hz, 8.6 Hz, 2H), 3.61 (t, J = 8.0 Hz, 2H), 1.82 (p, J = 7.8 Hz, 2H), 1.57 (p, J = 7.2 Hz, 2H), 1.40 (p, J = 6.9 Hz, 2H), 1.33-1.29 (m, 4H), .89 (t, J = 6.7 Hz, 3H) **^{13}C-NMR** (100 MHz, $CDCl_3$) δ: 148.9, 130.6, 129.8, 125.6, 125.0, 124.5, 32.0, 31.6, 29.3, 27.9, 22.8, 14.3 **HRMS (**EI) calc. for $C_{20}H_{23}N$ 277.1831, found 277.1817 **UV-Vis** (CH_2Cl_2) λ_{max} (ε, $cm^{-1}M^{-1}$) 255 (320000), 247 (sh,130000), 359 (190000), 388 (8500)

Preparation of 9-(4-phenylbutyl)acridine (5f):

The general procedure for the synthesis of 9-substituted acridine derivatives above was followed with 0.46 mL (3.0 mmol) 5-phenyl-1-pentyne. The reaction produced a pale green oil in 50 mg (10%) yield.

^{1}H-NMR (400 MHz, $CDCl_3$) δ: 8.22 (d, J = 8.7 Hz, 2H), 8.21 (d, J = 8.6 Hz, 2H), 7.76 (ddd, J = 8.4 Hz, .7 Hz, 8.6 Hz, 2H), 7.55 (ddd, J = 8.6 Hz, 10.3 Hz, 1.0 Hz, 2H), 7.28 (t, 2H), 7.21-7.16 (mp, 3H), 3.64 (t, J = 7.9 Hz, 2H), 2.70 (t, J = 6.9 Hz, 2H), 1.93-1.84 (brm, 4H) **^{13}C NMR** (100 MHz, $CDCl_3$) δ: 148.8, 142.1, 136.6, 129.9, 128.5, 126.0, 125.7, 125.0, 124.5, 35.8, 32.0, 31.11, 31.04, 27.7 **HRMS (**EI) calc. for $C_{23}H_{21}N$ 311.1674, found 311.1685 **UV-Vis** (CH_2Cl_2) λ_{max} (ε, $cm^{-1}M^{-1}$) 255 (89000), 247 (sh,40000), 360 (5900), 386 (2600)

Spectra for all molecules:
Bis(bromophenyl)amine (3):

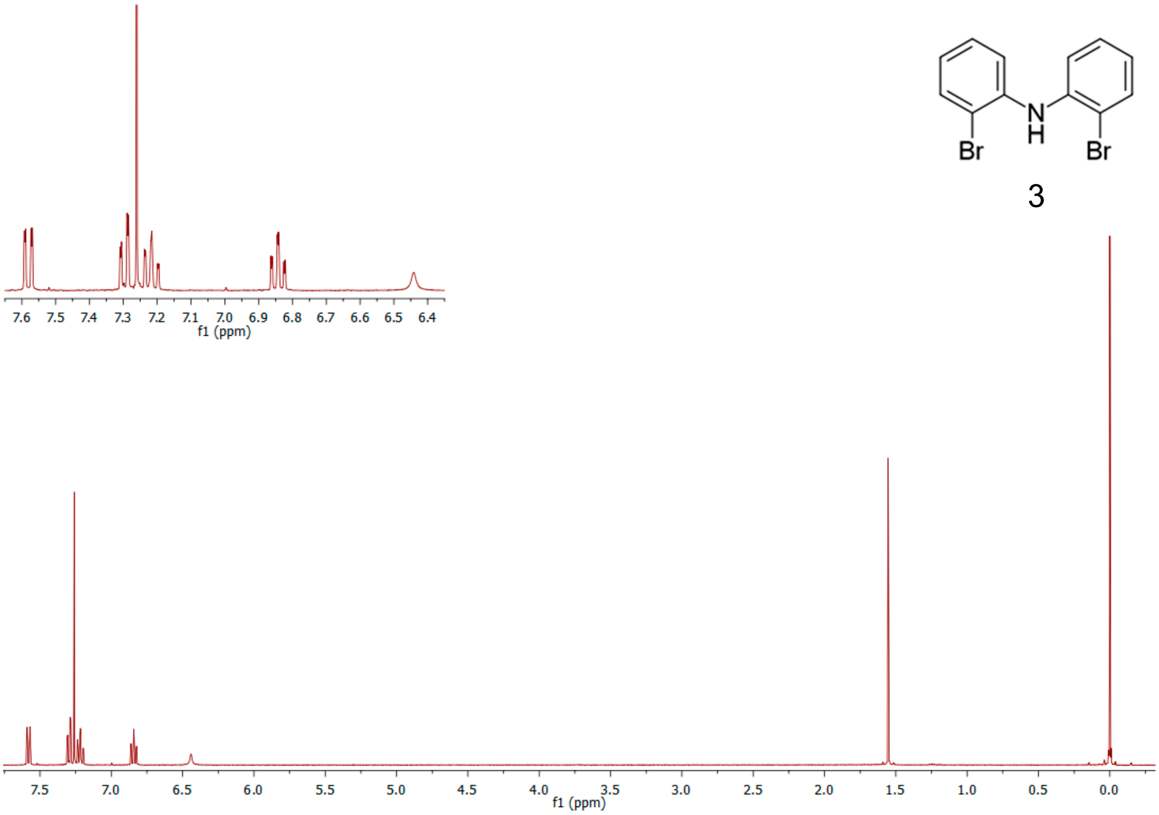

^{1}H-NMR (400 MHz, $CDCl_3$)

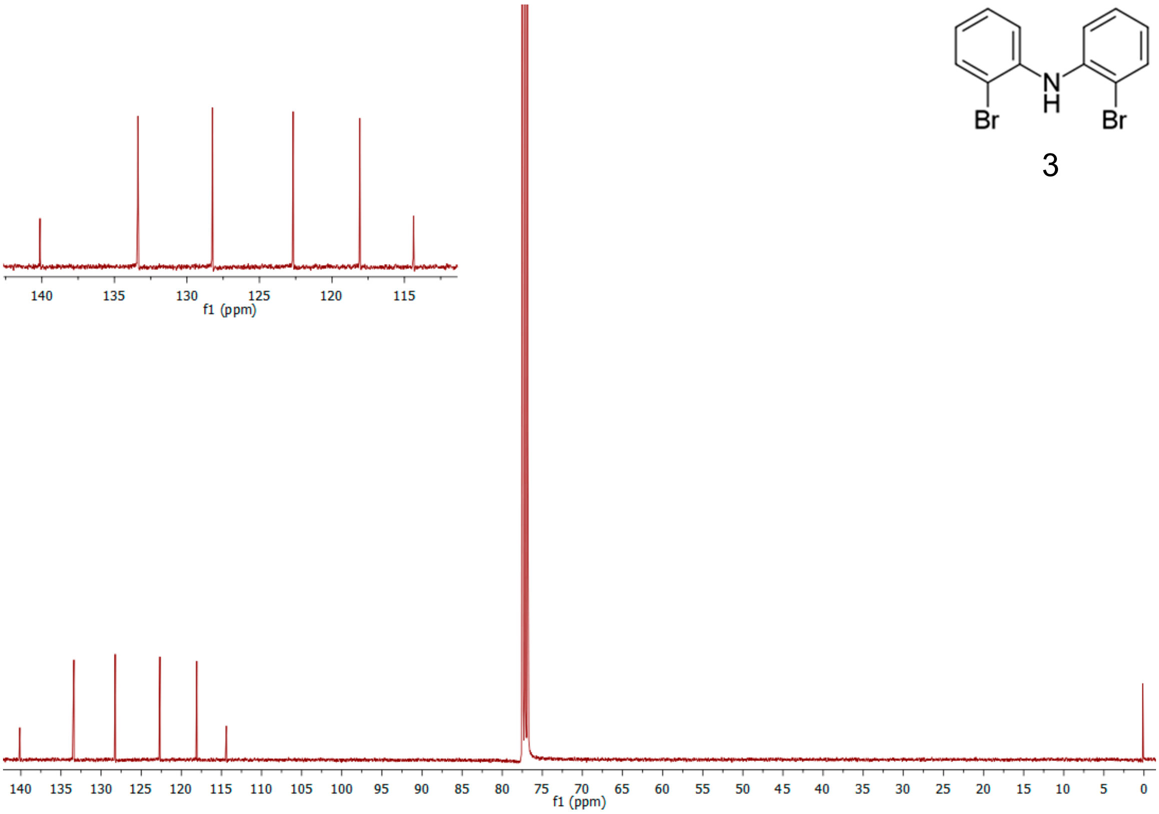

^{13}C-NMR (100 MHz, $CDCl_3$)

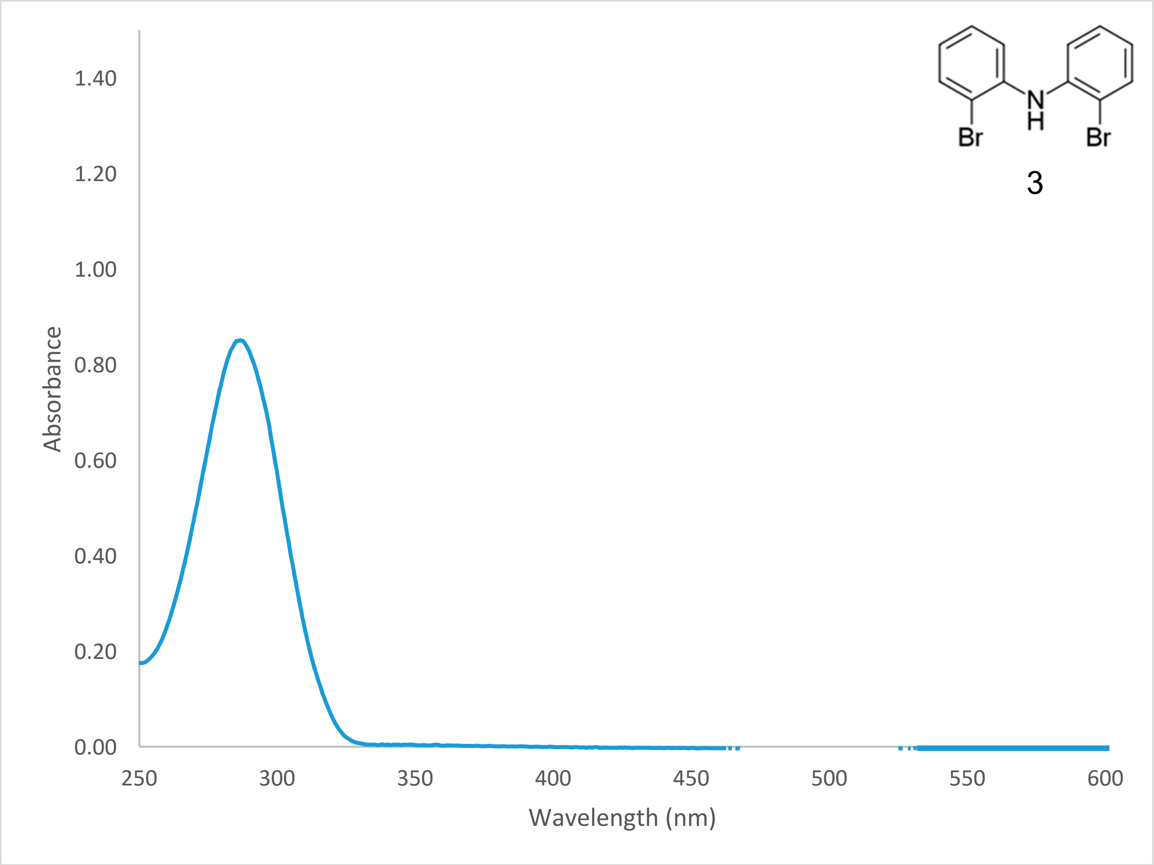
1.40
1.20
1.00
0.80
0.60
0.40
0.20
0.00
Absorbance
250
300
350
400
450
500
550
600
Wavelength (nm)
Br
N
H
Br
3

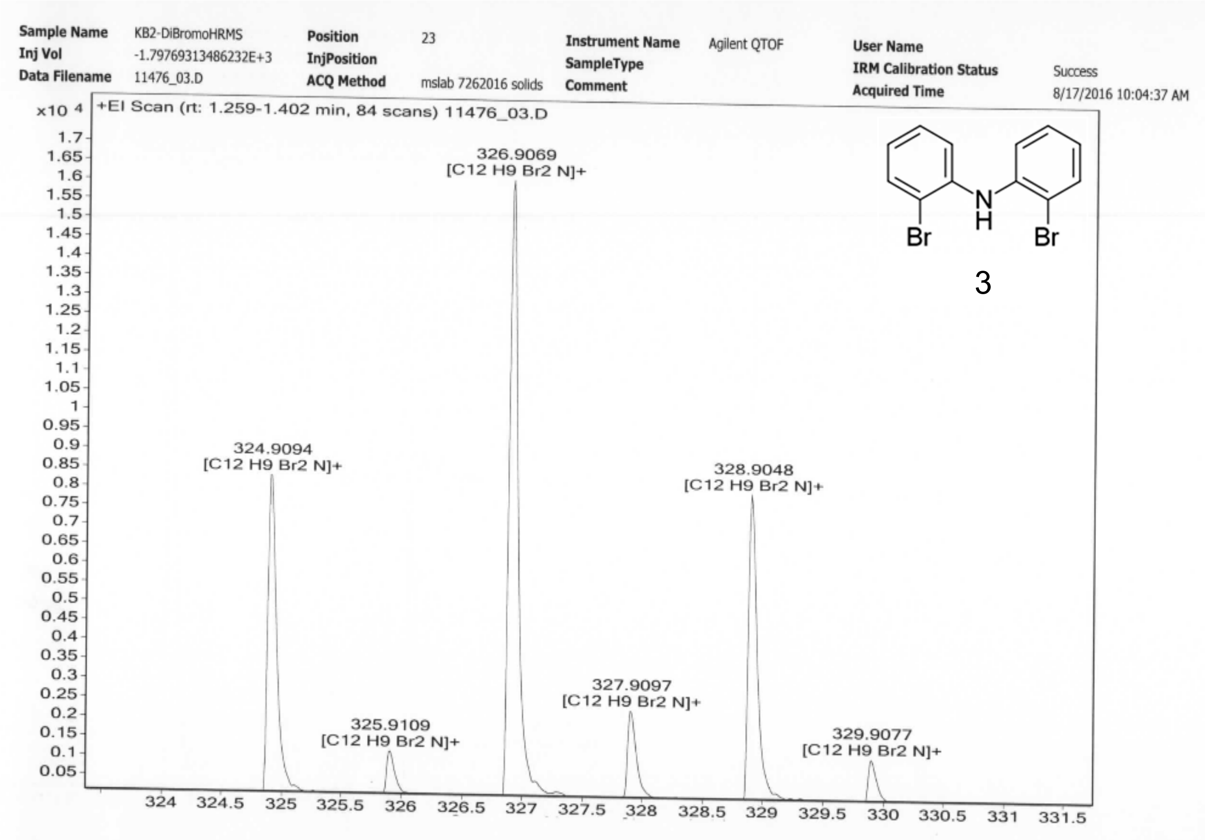
Sample Name KB2-DiBromoHRMS
Inj Vol -1.79769313486232E+3
Data Filename 11476_03.D
Position 23
InjPosition
ACQ Method mslab 7262016 solids
Instrument Name Agilent QTOF
SampleType
Comment
User Name
IRM Calibration Status Success
Acquired Time 8/17/2016 10:04:37 AM
x10 4
+EI Scan (rt: 1.259-1.402 min, 84 scans) 11476_03.D
326.9069
[C12 H9 Br2 N]+
324.9094
[C12 H9 Br2 N]+
328.9048
[C12 H9 Br2 N]+
325.9109
[C12 H9 Br2 N]+
327.9097
[C12 H9 Br2 N]+
329.9077
[C12 H9 Br2 N]+
Br
N
H
Br
3

Bis(tolan)amine (4a):

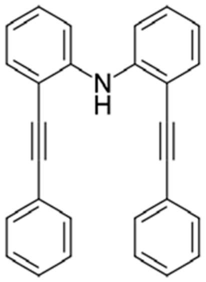

4a

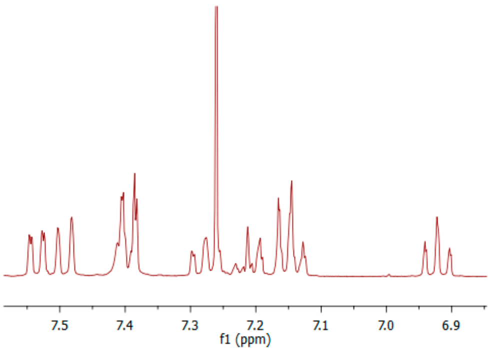

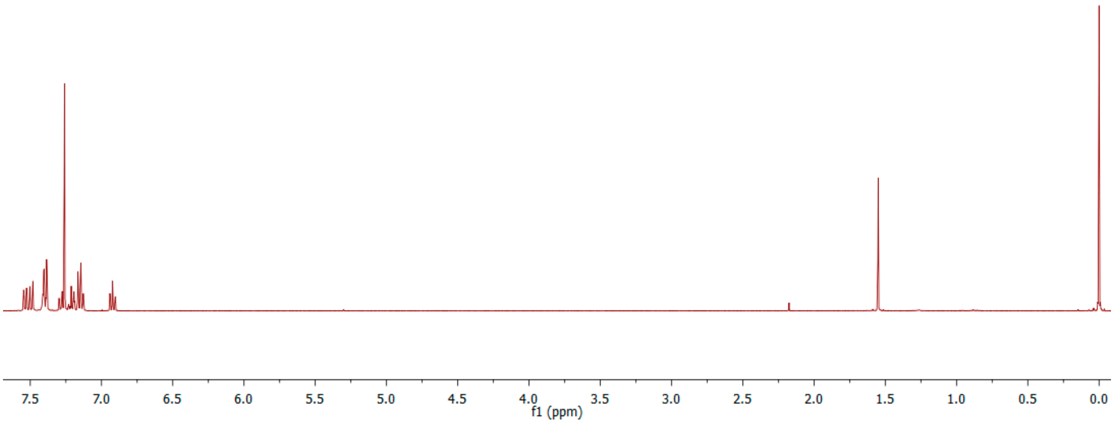

^{1}H-NMR (400 MHz, $CDCl_3$)

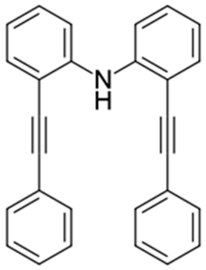

4a

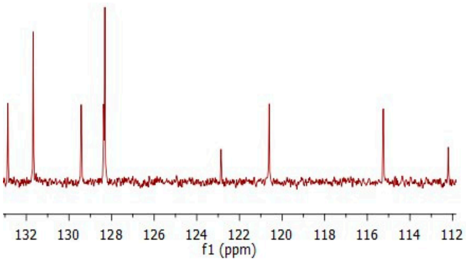

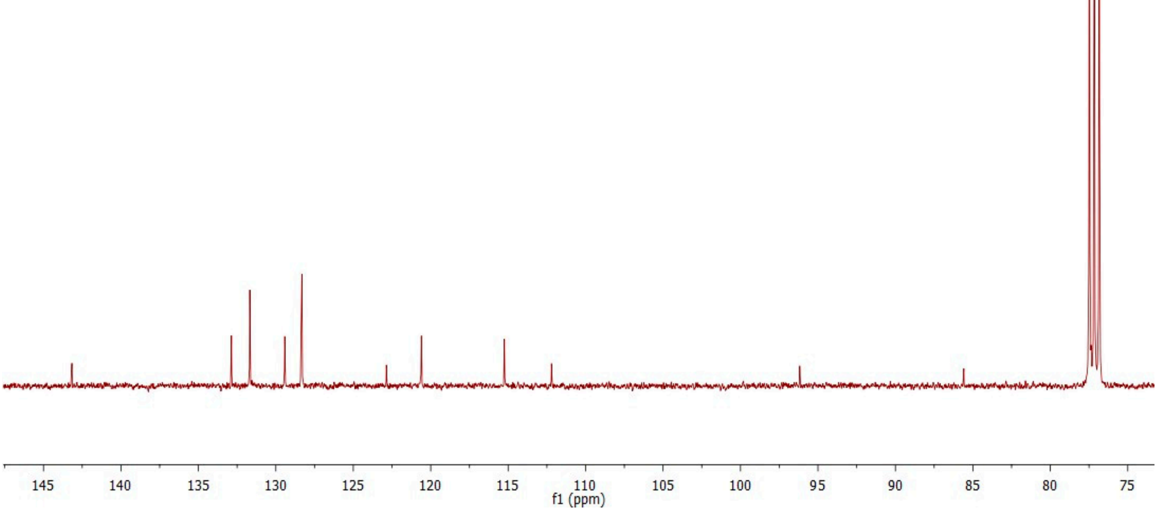

^{13}C-NMR (100 MHz, $CDCl_3$)

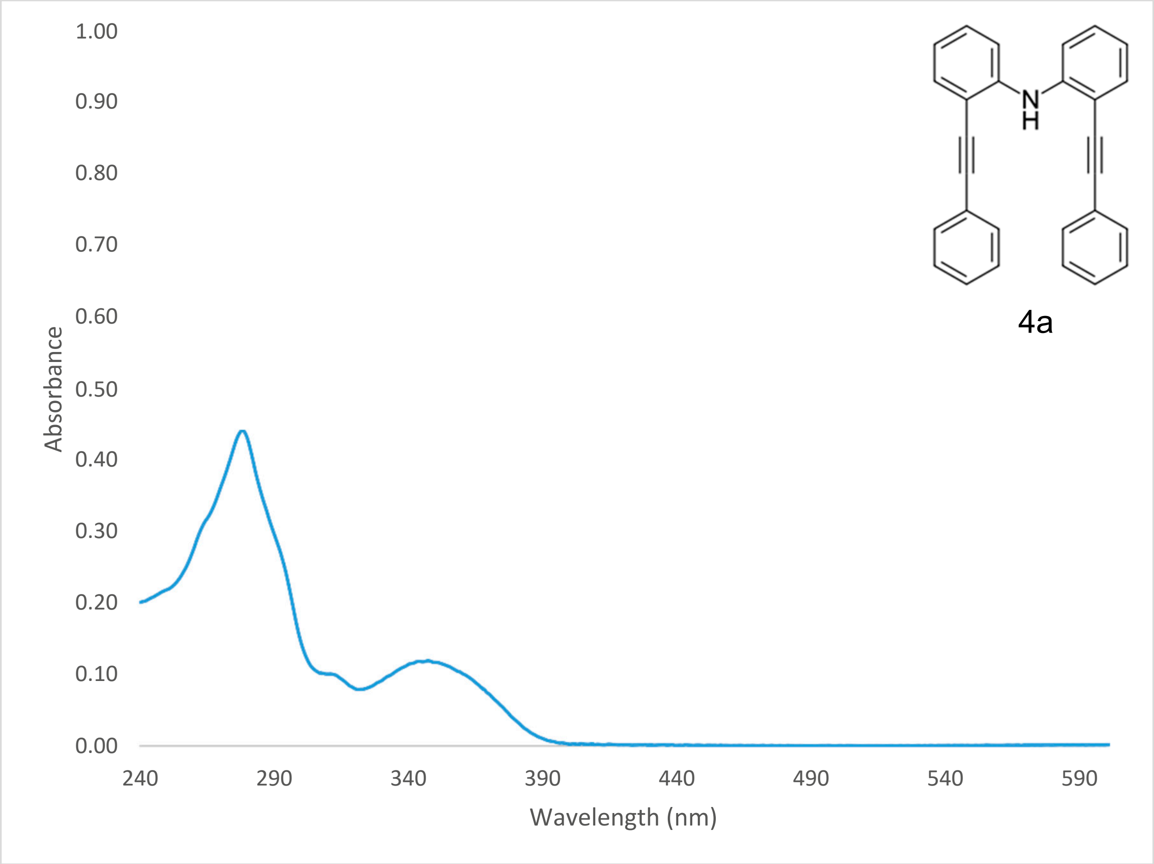

1.00
0.90
0.80
0.70
0.60
0.50
0.40
0.30
0.20
0.10
0.00
Absorbance
240
290
340
390
440
490
540
590
Wavelength (nm)
N
H
4a

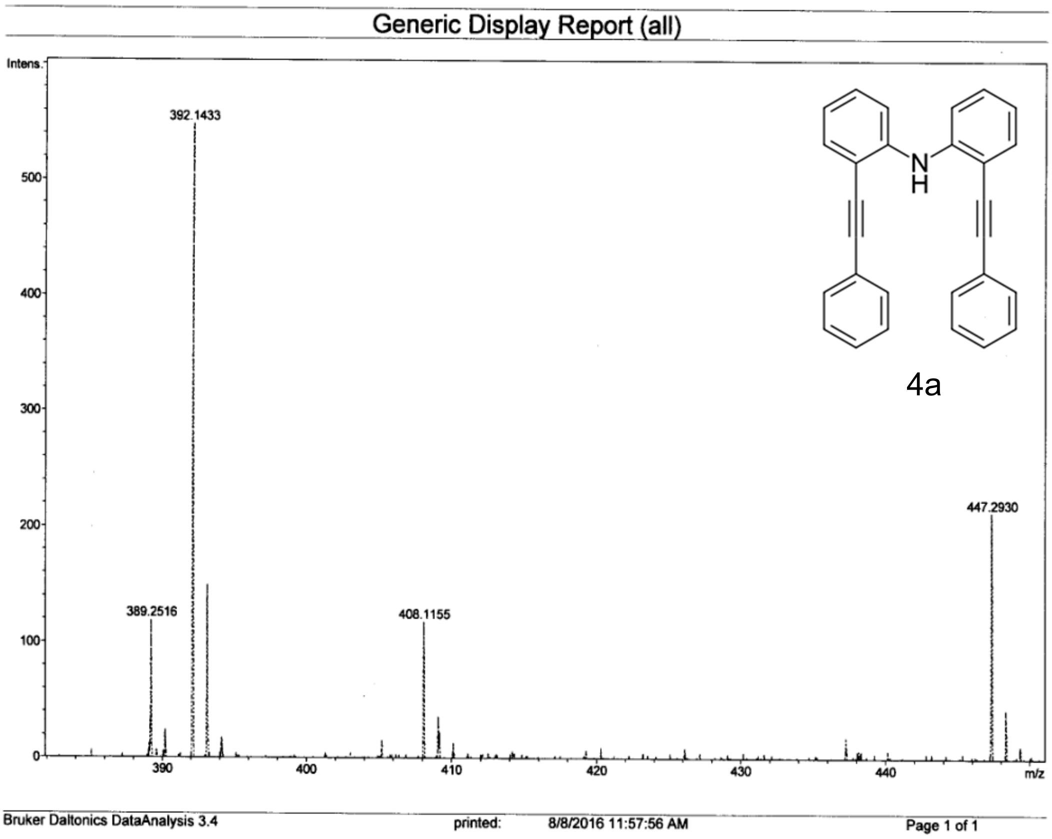

Generic Display Report (all)
Intens.
500
400
300
200
100
0
392.1433
389.2516
408.1155
447.2930
390
400
410
420
430
440
m/z
N
H
4a
Bruker Daltonics DataAnalysis 3.4
printed: 8/8/2016 11:57:56 AM
Page 1 of 1

9-(Benzyl)acridine (5a):

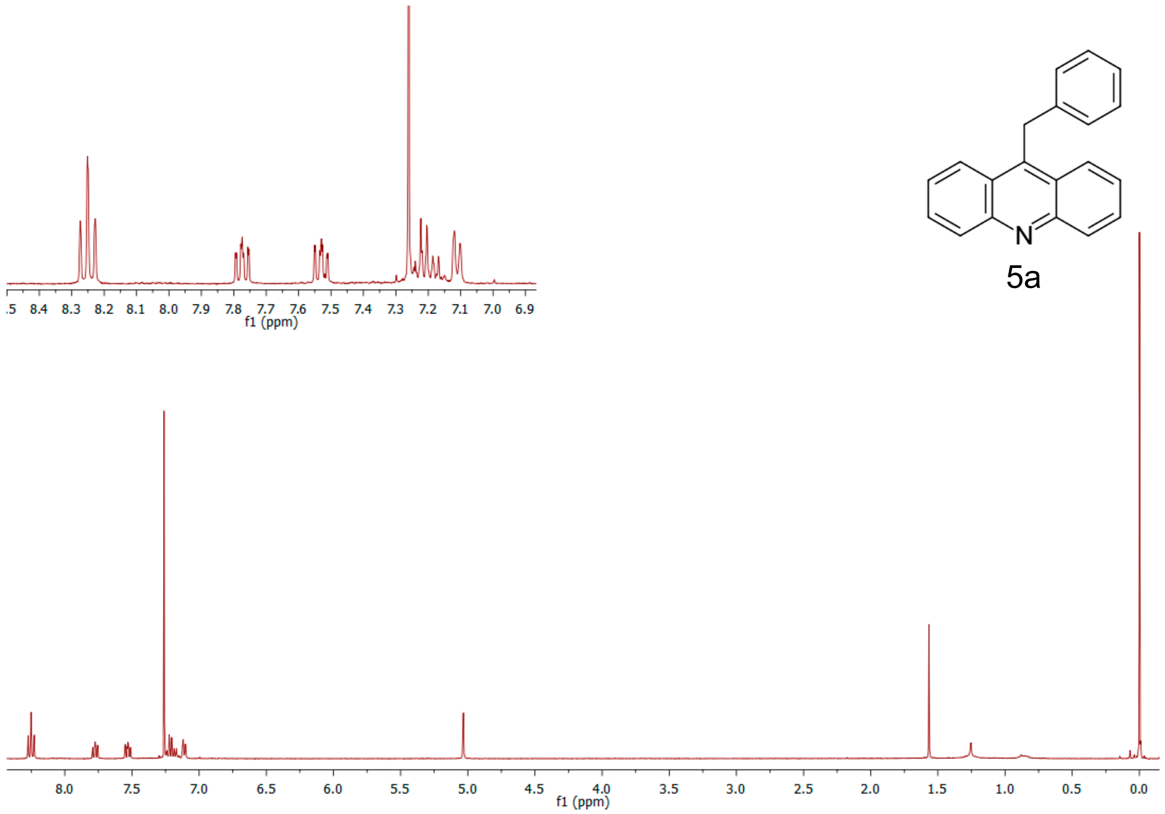

^{1}H-NMR (400 MHz, $CDCl_3$)

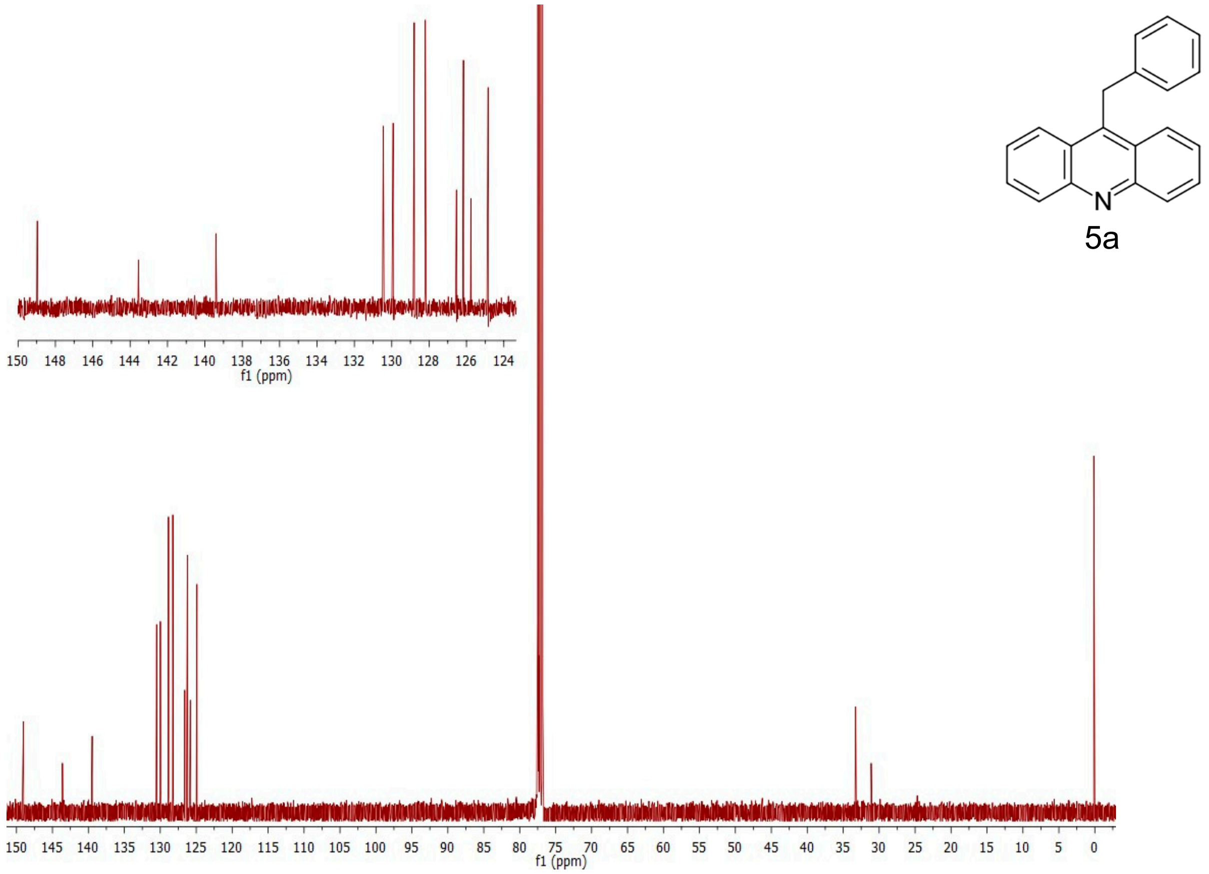

^{13}C-NMR (100 MHz, $CDCl_3$)

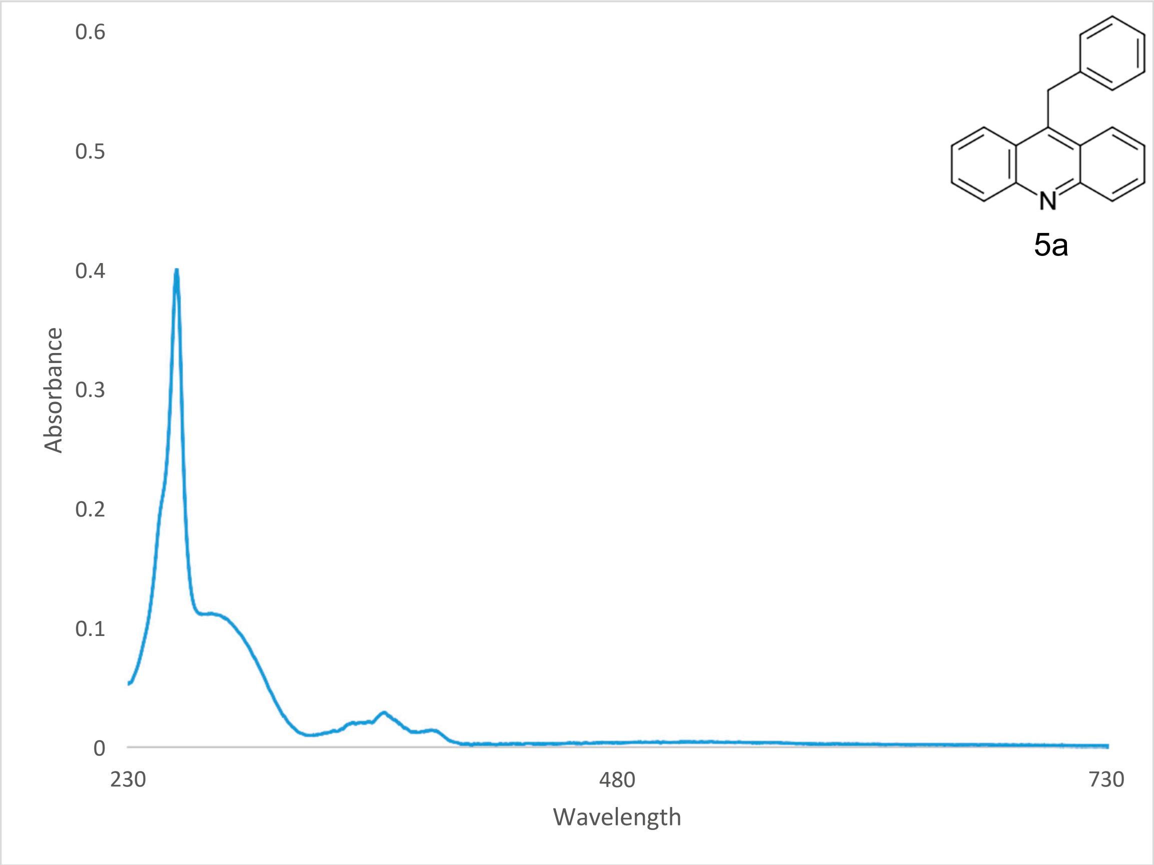
0.6
0.5
0.4
0.3
0.2
0.1
0
Absorbance
230
480
730
Wavelength
N
5a

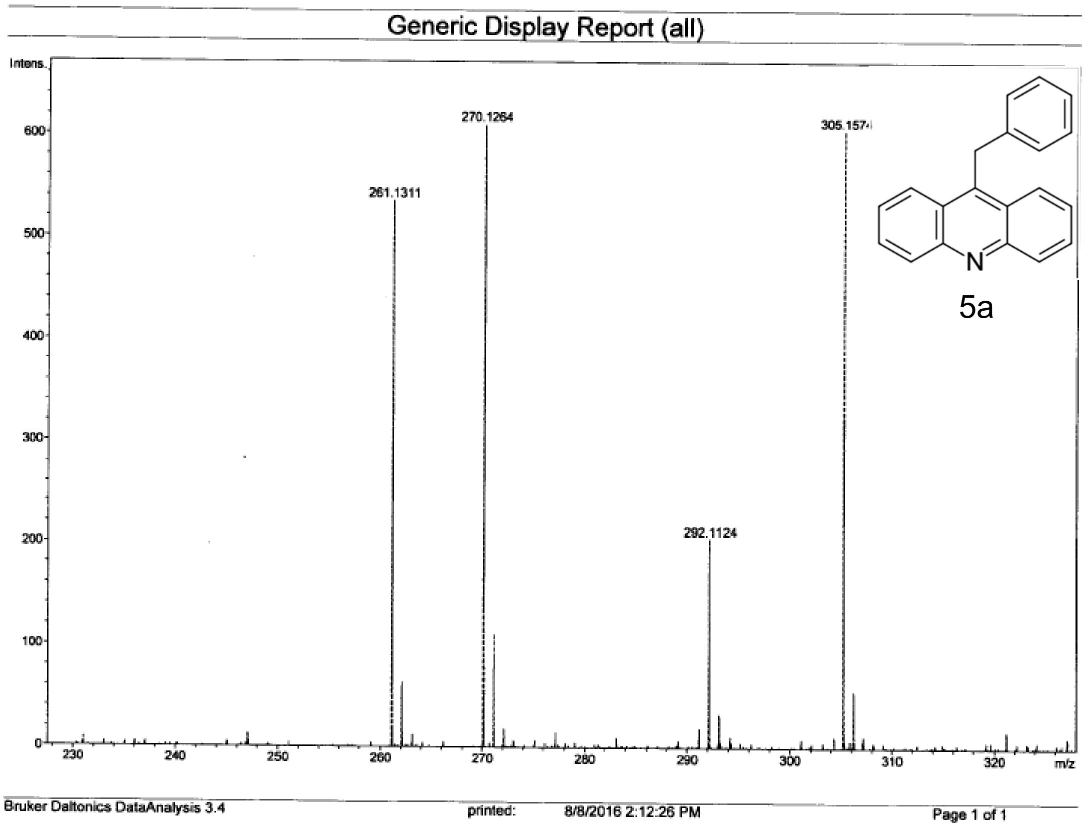
Generic Display Report (all)
Intens.
600
500
400
300
200
100
0
261.1311
270.1264
292.1124
305.1574
230
240
250
260
270
280
290
300
310
320
m/z
N
5a
Bruker Daltonics DataAnalysis 3.4
printed: 8/8/2016 2:12:26 PM
Page 1 of 1

9-(4-Methylbenzyl)acridine (5b):

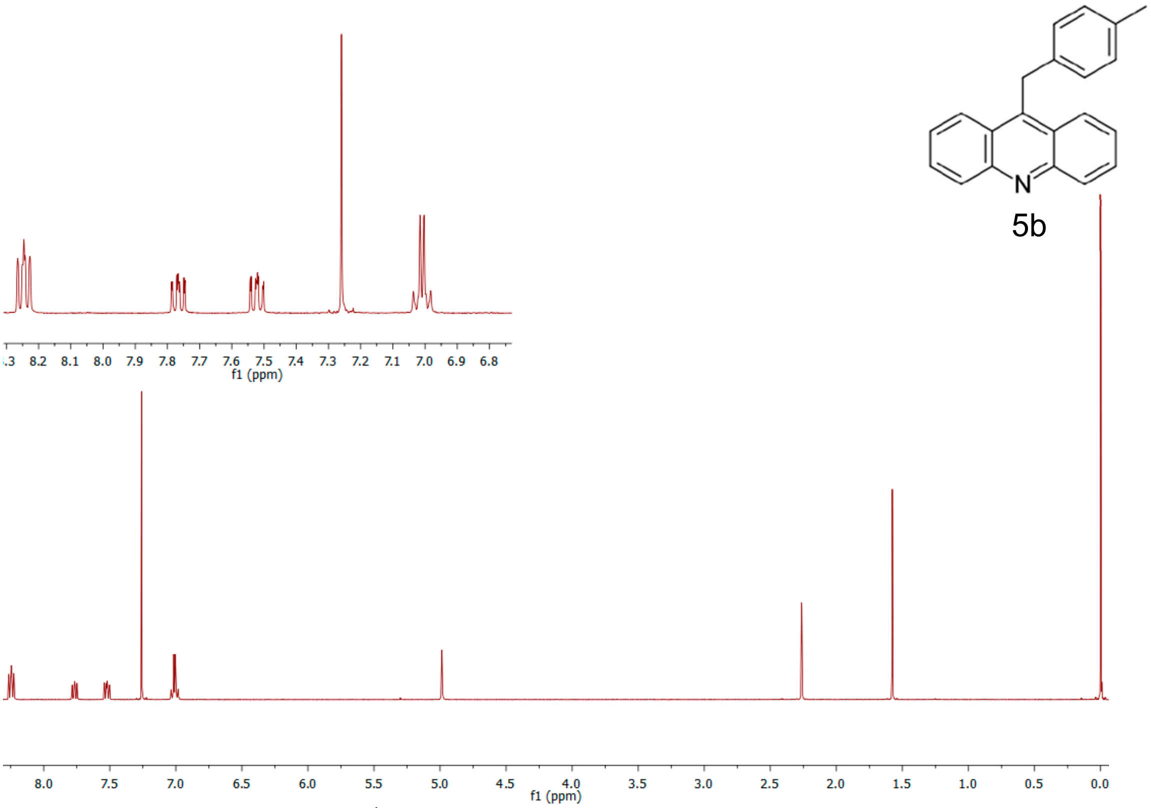

^{1}H-NMR (400 MHz, $CDCl_3$)

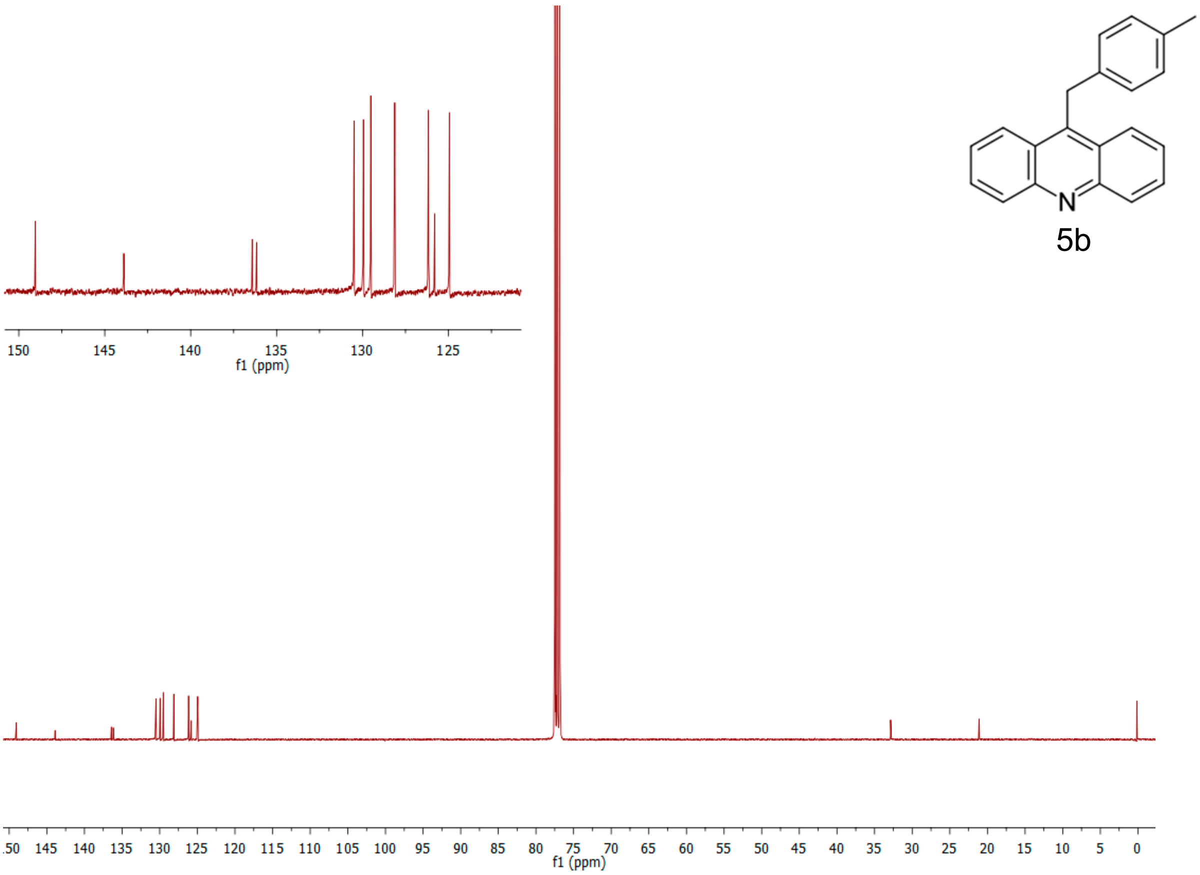

^{13}C-NMR (100 MHz, $CDCl_3$)

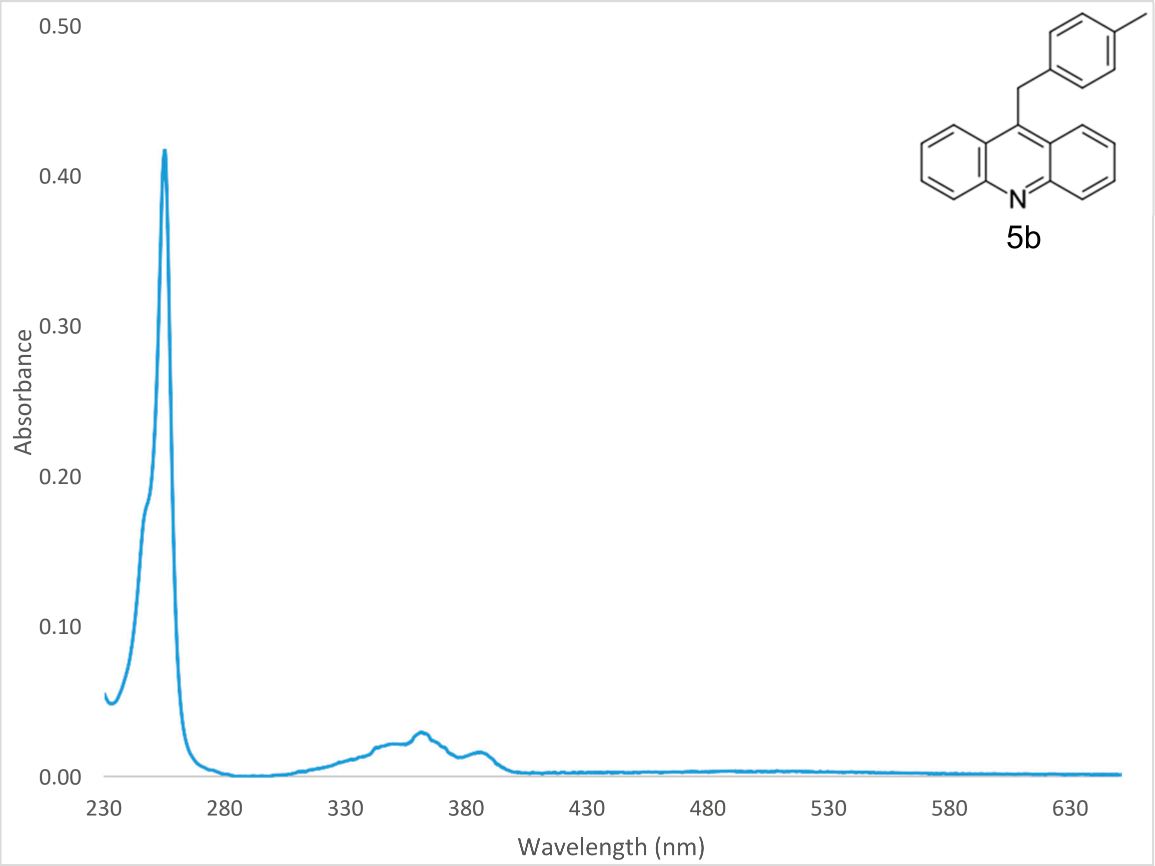
0.50
0.40
0.30
0.20
0.10
0.00
Absorbance
230
280
330
380
430
480
530
580
630
Wavelength (nm)
N
5b

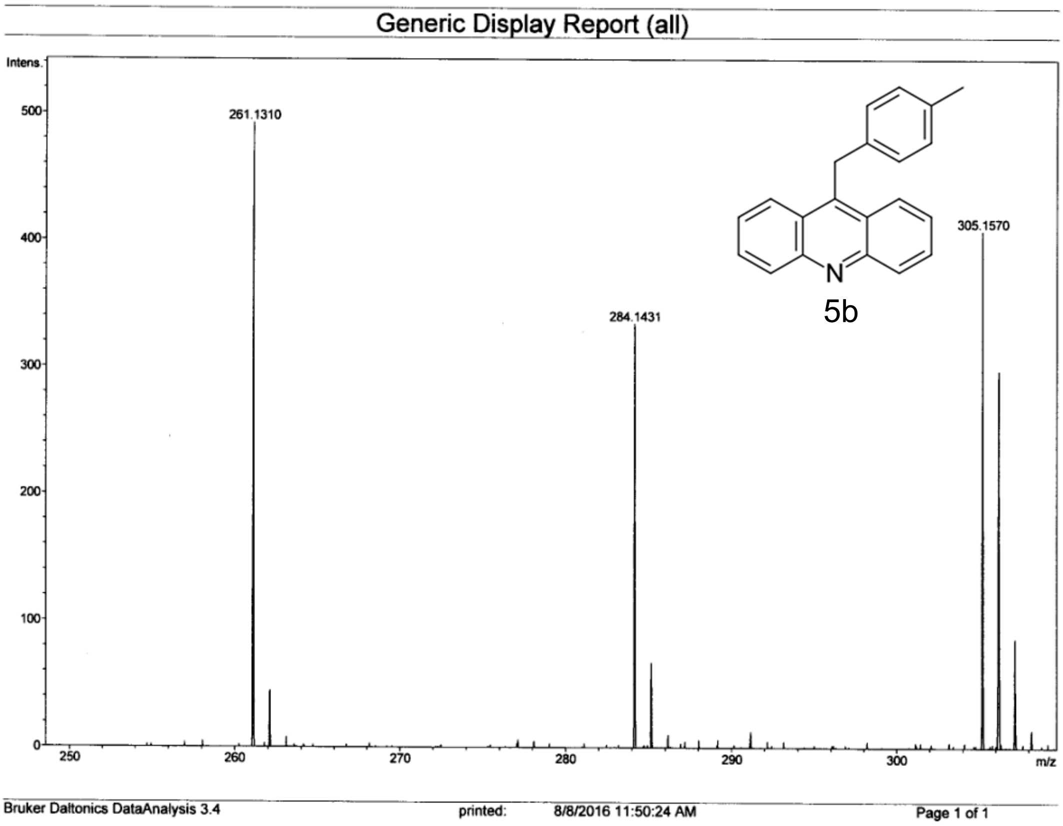
Generic Display Report (all)
Intens.
500
400
300
200
100
0
261.1310
284.1431
305.1570
N
5b
250
260
270
280
290
300
m/z
Bruker Daltonics DataAnalysis 3.4
printed: 8/8/2016 11:50:24 AM
Page 1 of 1

9-(4-Methoxybenzyl)acridine (5c):

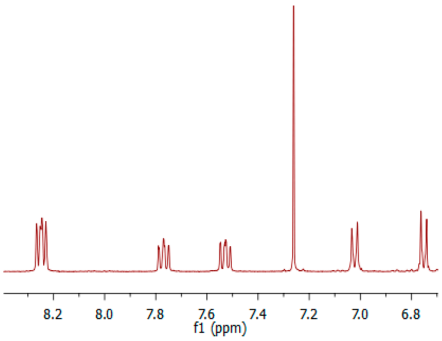

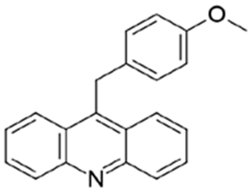

5c

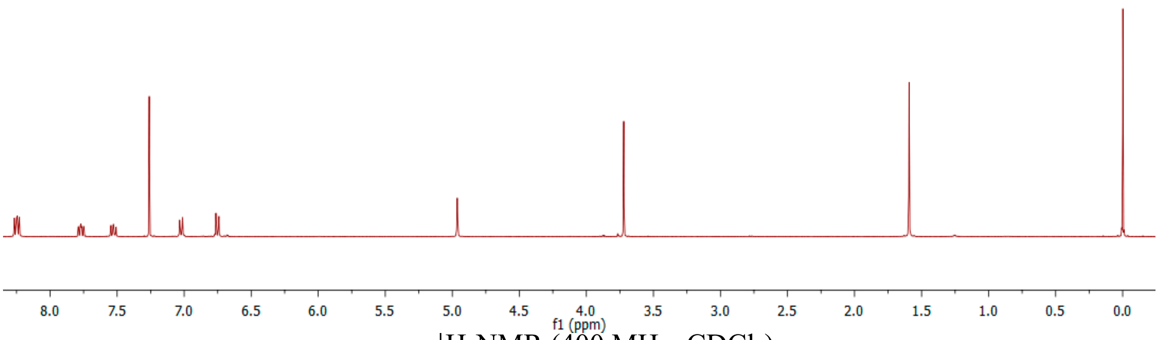

^{1}H-NMR (400 MHz, $CDCl_3$)

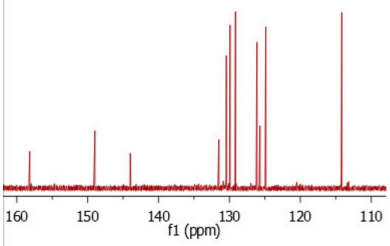

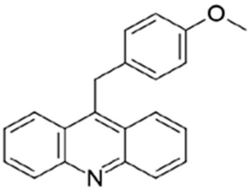

5c

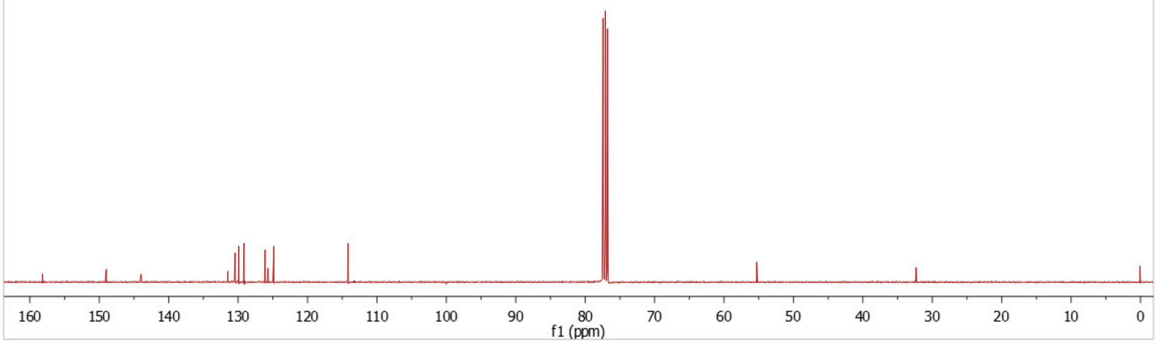

^{13}C-NMR (100 MHz, $CDCl_3$)

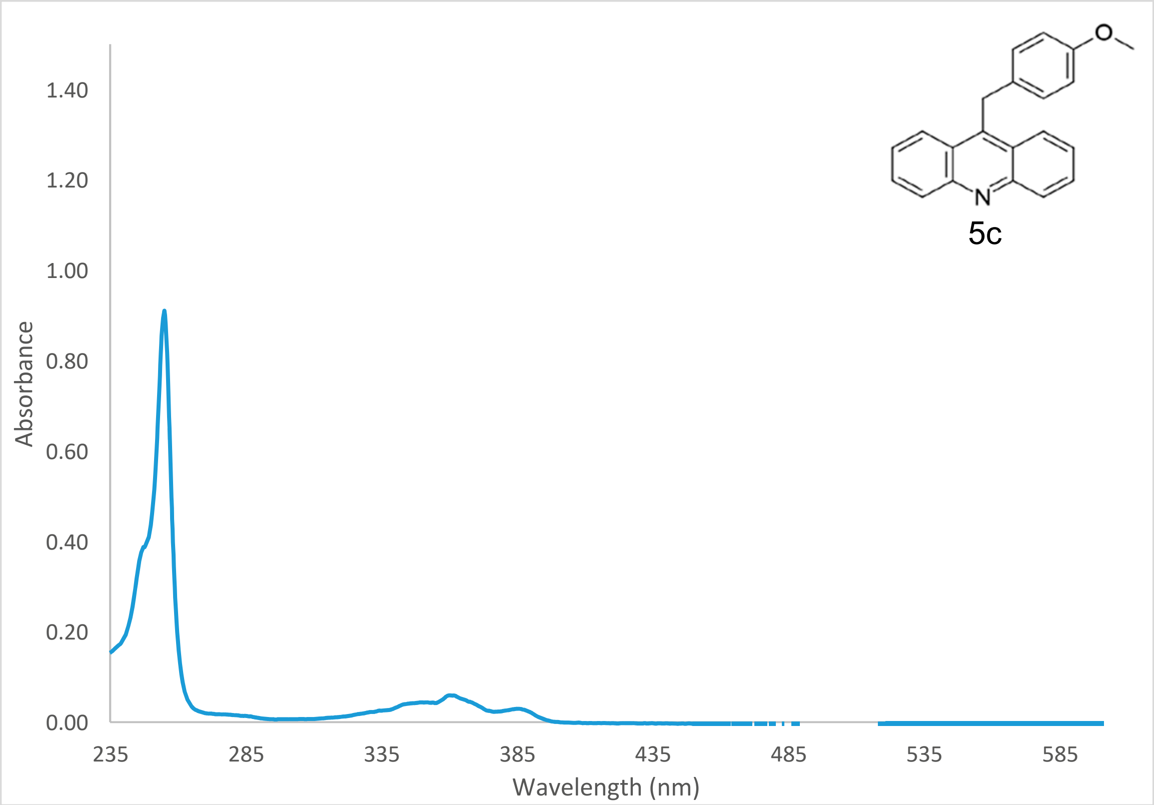
1.40
1.20
1.00
0.80
0.60
0.40
0.20
0.00
Absorbance
235
285
335
385
435
485
535
585
Wavelength (nm)
N
5c

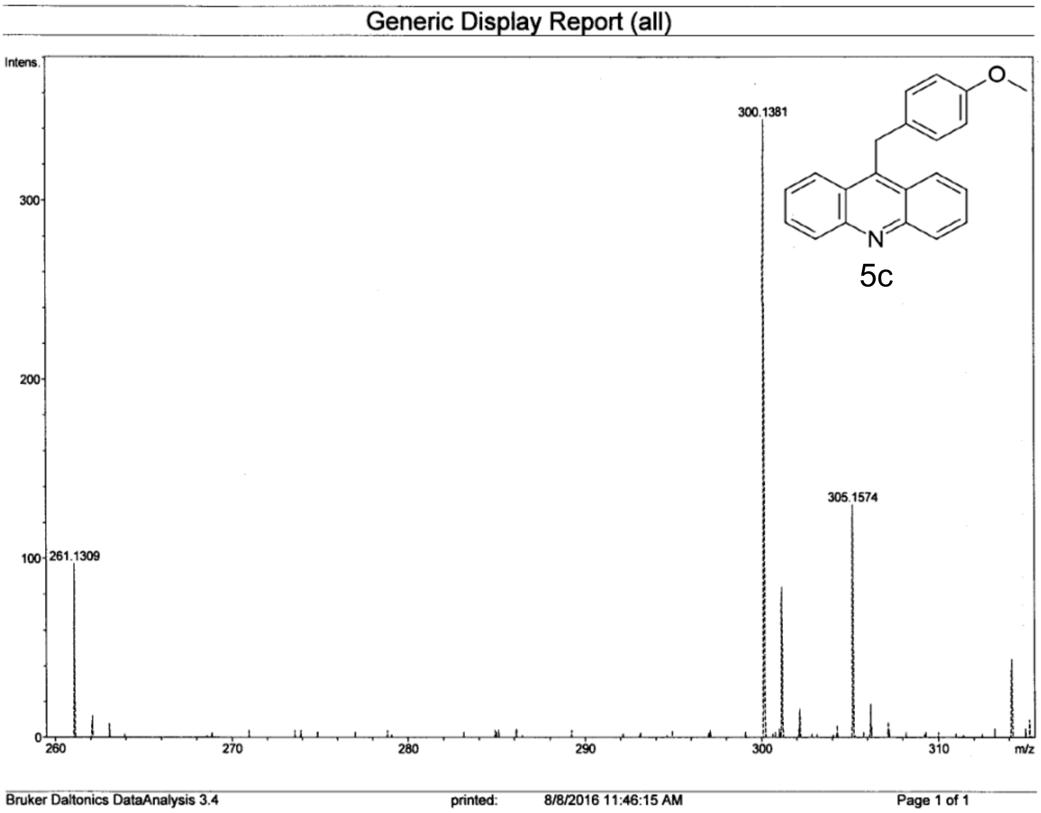
Generic Display Report (all)
Intens.
300
200
100
0
300.1381
305.1574
261.1309
260
270
280
290
300
310
m/z
N
5c
Bruker Daltonics DataAnalysis 3.4
printed: 8/8/2016 11:46:15 AM
Page 1 of 1

9-(4-Fluorobenzyl)acridine (5d):

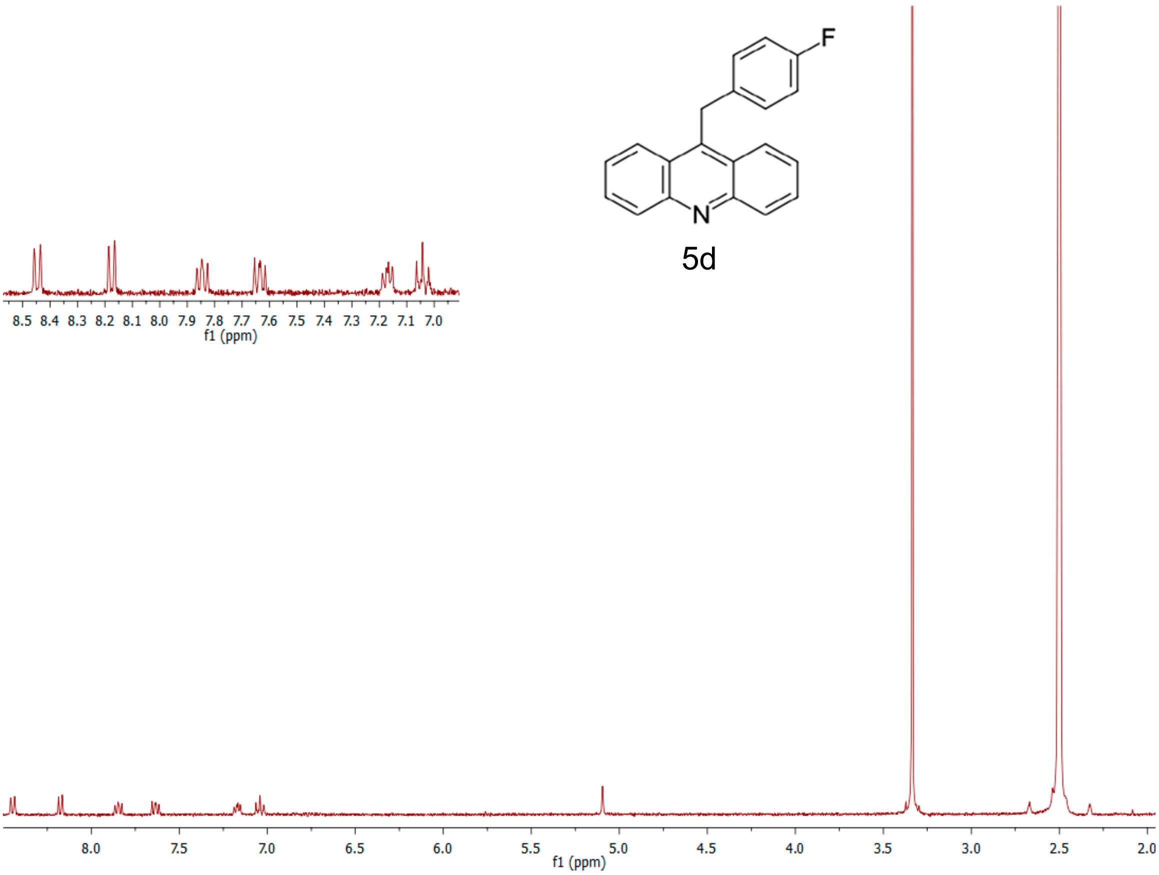

^{1}H-NMR (400 MHz, DMSO)

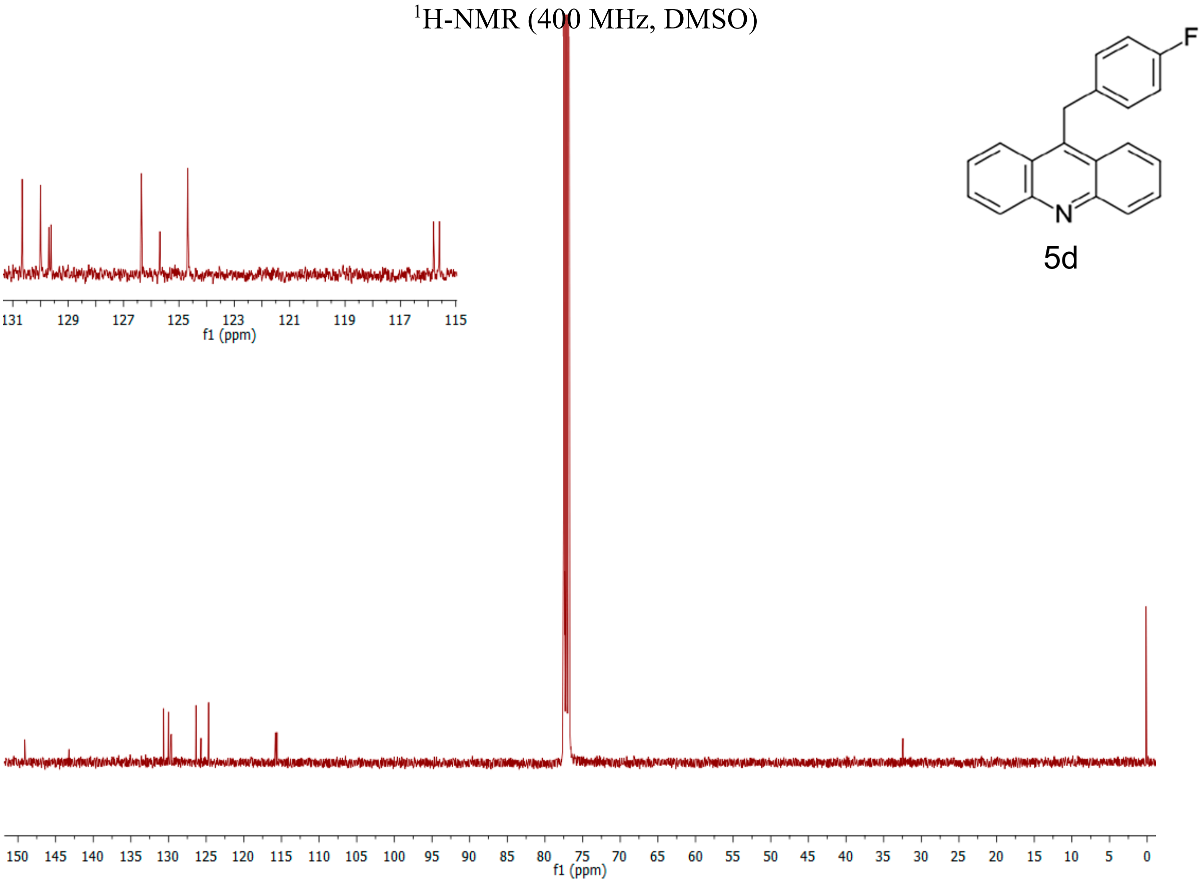

^{13}C-NMR (100 MHz, $CDCl_3$)

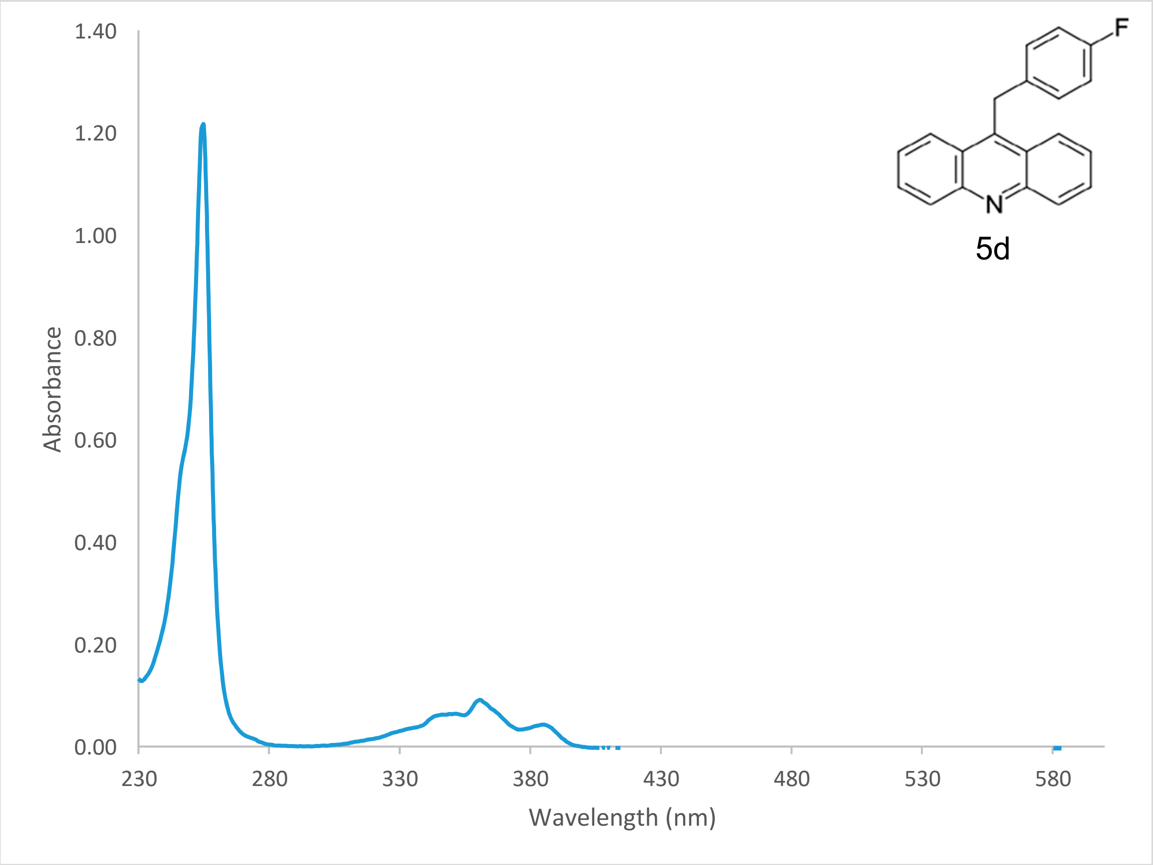

Absorbance
1.40
1.20
1.00
0.80
0.60
0.40
0.20
0.00
230
280
330
380
430
480
530
580
Wavelength (nm)
F
N
5d

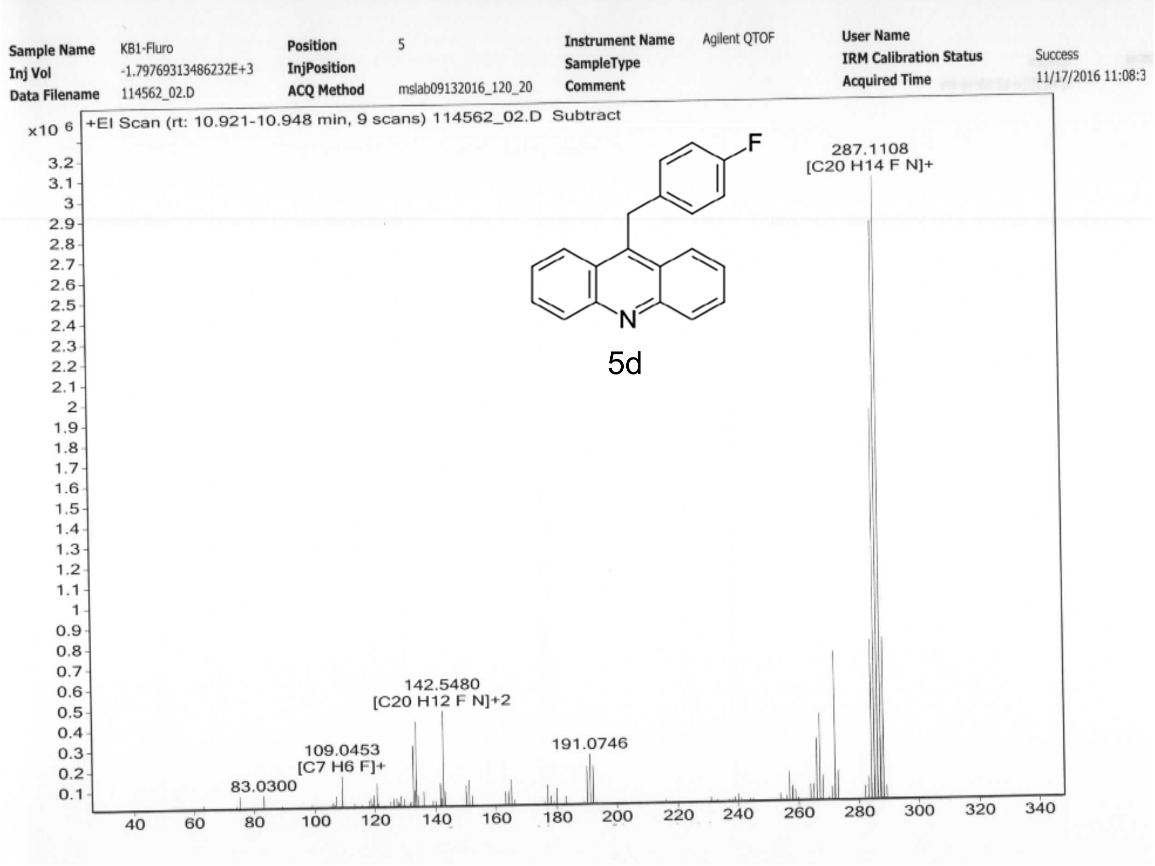

Sample Name KB1-Fluro
Inj Vol -1.79769313486232E+3
Data Filename 114562_02.D
Position 5
InjPosition
ACQ Method mslab09132016_120_20
Instrument Name Agilent QTOF
SampleType
Comment
User Name
IRM Calibration Status Success
Acquired Time 11/17/2016 11:08:3
x10 6
+EI Scan (rt: 10.921-10.948 min, 9 scans) 114562_02.D Subtract
287.1108
[C20 H14 F N]+
142.5480
[C20 H12 F N]+2
109.0453
[C7 H6 F]+
191.0746
83.0300
F
N
5d

9-Heptylacridine (5e):

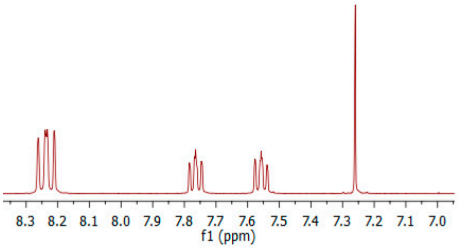

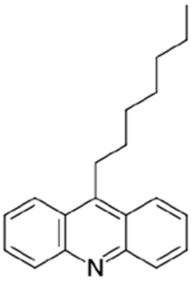

5e

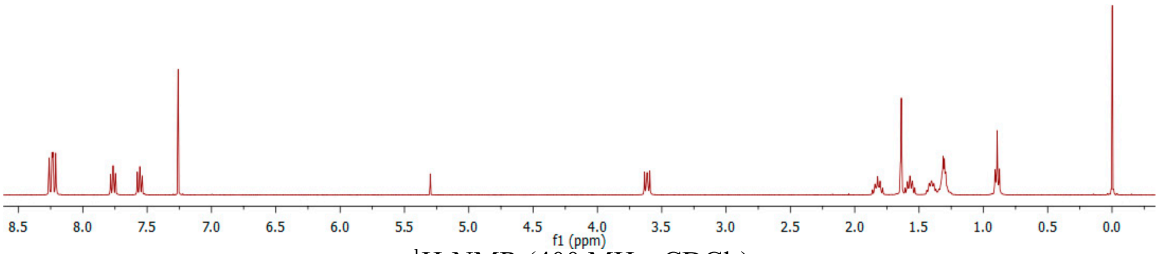

^{1}H-NMR (400 MHz, $CDCl_3$)

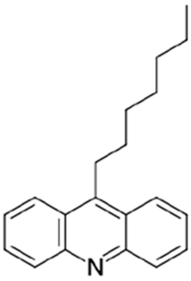

5e

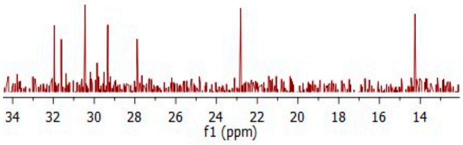

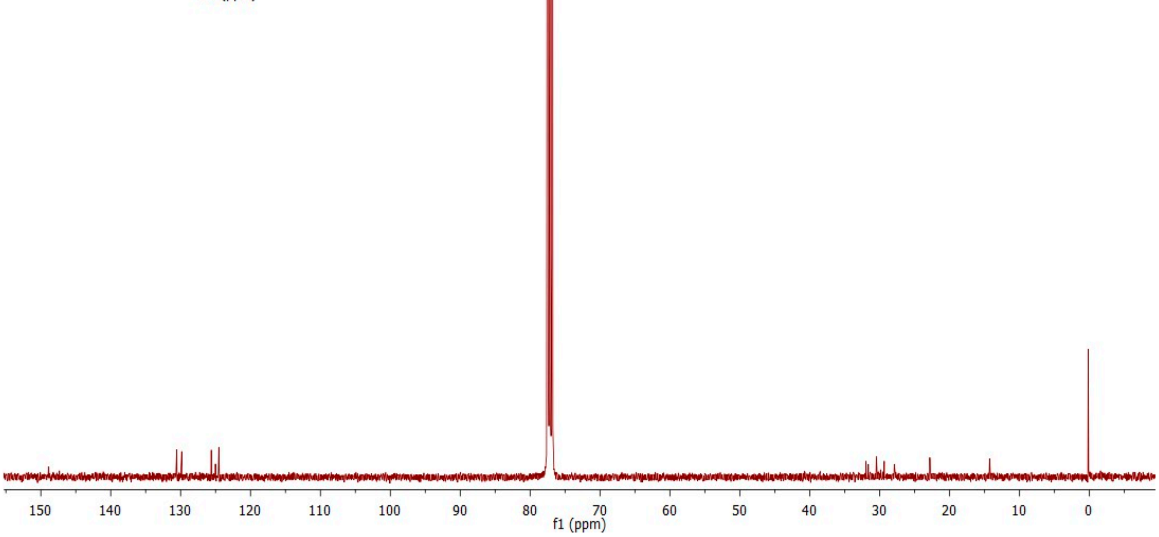

^{13}C-NMR (100 MHz, $CDCl_3$)

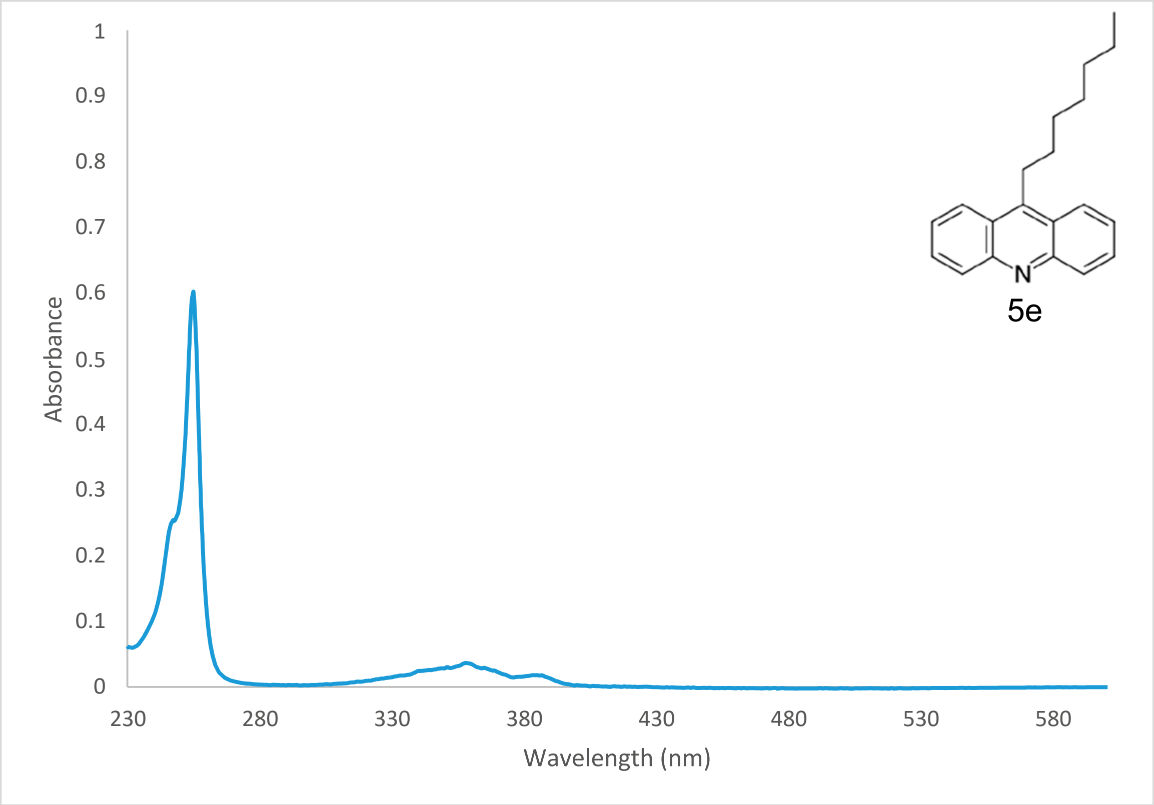

Absorbance
Wavelength (nm)
5e
N

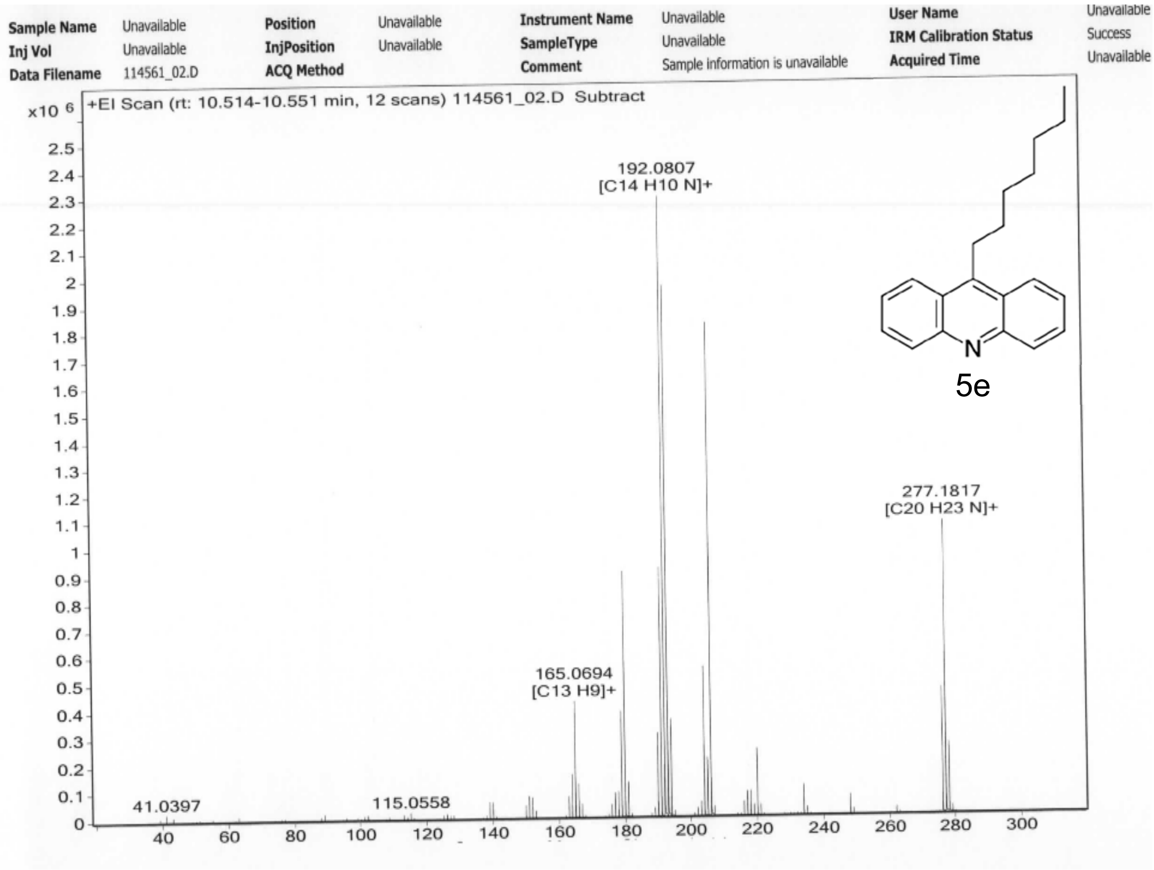

Sample Name Unavailable
Inj Vol Unavailable
Data Filename 114561_02.D
Position Unavailable
InjPosition Unavailable
ACQ Method
Instrument Name Unavailable
SampleType Unavailable
Comment Sample information is unavailable
User Name Unavailable
IRM Calibration Status Success
Acquired Time Unavailable
x10 6 +EI Scan (rt: 10.514-10.551 min, 12 scans) 114561_02.D Subtract
192.0807
[C14 H10 N]+
165.0694
[C13 H9]+
277.1817
[C20 H23 N]+
41.0397
115.0558
5e
N

9-(4-Phenylbutyl)acridine (5f):

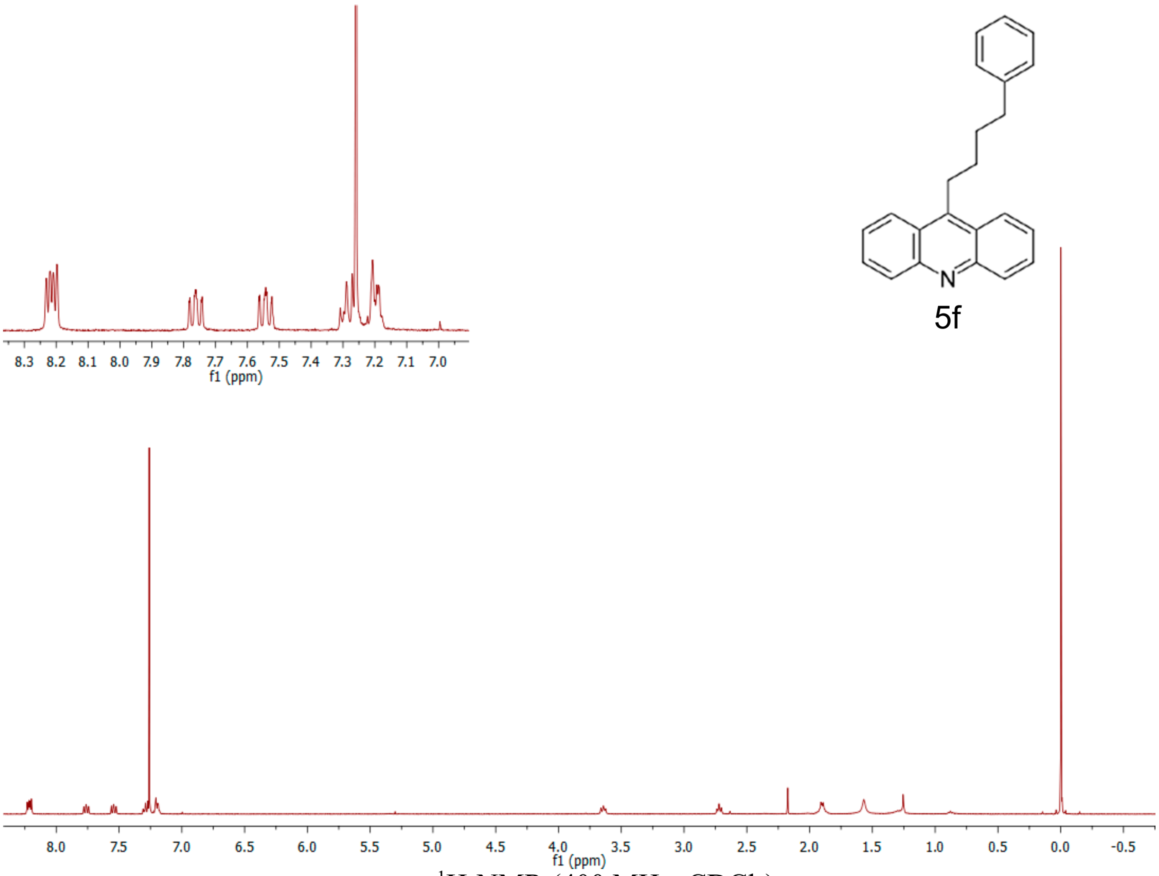

^{1}H-NMR (400 MHz, $CDCl_3$)

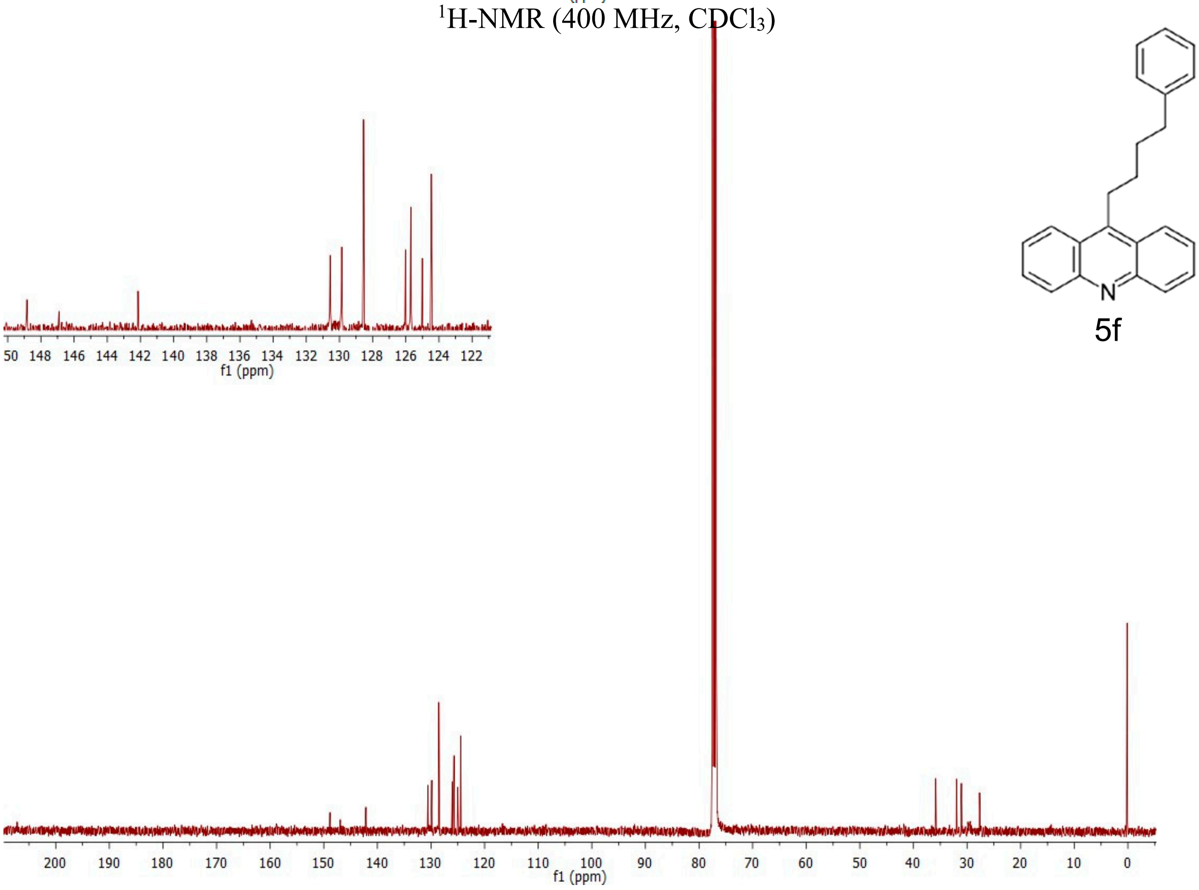

^{13}C-NMR (100 MHz, $CDCl_3$)

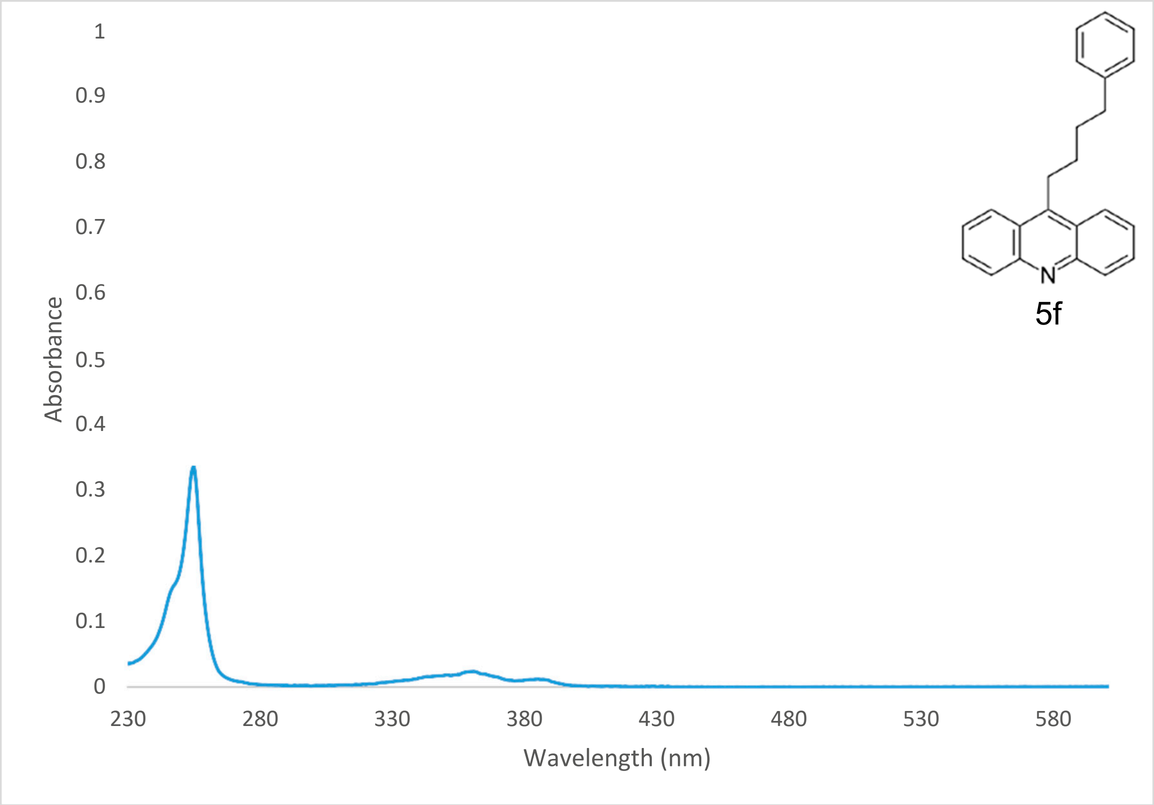
Absorbance
Wavelength (nm)
1
0.9
0.8
0.7
0.6
0.5
0.4
0.3
0.2
0.1
0
230
280
330
380
430
480
530
580
N
5f

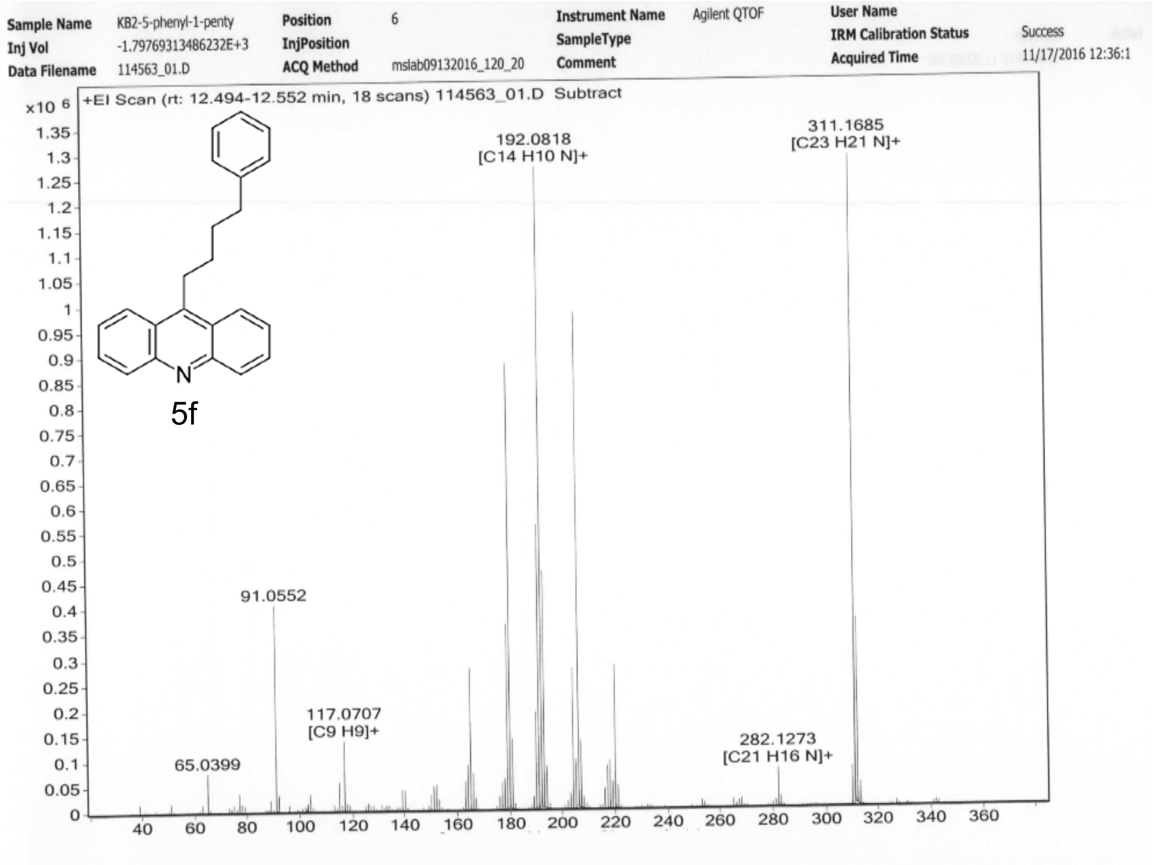
Sample Name KB2-5-phenyl-1-penty
Inj Vol -1.79769313486232E+3
Data Filename 114563_01.D
Position 6
InjPosition
ACQ Method mslab09132016_120_20
Instrument Name Agilent QTOF
SampleType
Comment
User Name
IRM Calibration Status Success
Acquired Time 11/17/2016 12:36:1
x10 6
+EI Scan (rt: 12.494-12.552 min, 18 scans) 114563_01.D Subtract
192.0818
[C14 H10 N]+
311.1685
[C23 H21 N]+
91.0552
117.0707
[C9 H9]+
65.0399
282.1273
[C21 H16 N]+
N
5f
